# Unraveling the mysterious failure of Cu/SAPO-34 selective catalytic reduction catalysts

**DOI:** 10.1038/s41467-019-09021-3

**Published:** 2019-03-08

**Authors:** Aiyong Wang, Ying Chen, Eric D. Walter, Nancy M. Washton, Donghai Mei, Tamas Varga, Yilin Wang, János Szanyi, Yong Wang, Charles H. F. Peden, Feng Gao

**Affiliations:** 1 Institute for Integrated Catalysis, Pacific Northwest National Laboratory, P.O. Box 999, Richland, WA 99354 USA; 2 Environmental Molecular Sciences Laboratory, Pacific Northwest National Laboratory, P.O. Box 999, Richland, WA 99354 USA

## Abstract

Commercial Cu/SAPO-34 selective catalytic reduction (SCR) catalysts have experienced unexpected and quite perplexing failure. Understanding the causes at an atomic level is vital for the synthesis of more robust Cu/SAPO-34 catalysts. Here we show, via application of model catalysts with homogeneously dispersed isolated Cu ions, that Cu transformations resulting from low-temperature hydrothermal aging and ambient temperature storage can be semi-quantitatively probed with 2-dimensional pulsed electron paramagnetic resonance. Coupled with kinetics, additional material characterizations and DFT simulations, we propose the following catalyst deactivation steps: (1) detachment of Cu(II) ions from cationic positions in the form of Cu(OH)_2_; (2) irreversible hydrolysis of the SAPO-34 framework forming terminal Al species; and (3) interaction between Cu(OH)_2_ and terminal Al species forming SCR inactive, Cu-aluminate like species. Especially significant is that these reactions are greatly facilitated by condensed water molecules under wet ambient conditions, causing low temperature failure of the commercial Cu/SAPO-34 catalysts.

## Abstract (web summary)

Understanding the failure of commercial Cu/SAPO-34 selective catalytic reduction (SCR) catalysts at an atomic level is vital for the synthesis of more robust catalysts. Here the authors unravel the mysterious failure of Cu/SAPO-34 SCR catalysts via application of model catalysts and 2-dimensional pulsed electron paramagnetic resonance

## Introduction

The commercialization of hydrothermally robust, copper-exchanged small pore molecular sieve materials, Cu/SSZ-13 and Cu/SAPO-34, as selective catalytic reduction (SCR) catalysts for diesel engine exhaust aftertreatment, has been a major milestone in environmental catalysis in recent years^1–3^. Approaching the end of the first decade of commercial use, however, these two catalysts are now facing significantly different fates. While Cu/SSZ-13 continues to provide satisfactory performance, Cu/SAPO-34 is gradually retreating from the market due to performance failures. This was unexpected in light of the fact that Cu/SAPO-34 has been repeatedly found to be more stable than Cu/SSZ-13 during accelerated high-temperature (>700 °C) hydrothermal aging^4–6^, which generally translates to better long-term stability for a SCR catalyst.

Recent investigations indicate that Cu/SAPO-34 lacks durability at low temperatures (<100 °C) in the presence of moisture, with causes that are still not clearly understood^6–8^. Through our own investigations with the current state-of-the-art Cu/SAPO-34 catalysts, and from recent literature reports^6,7^, the unexpected low-temperature Cu/SAPO-34 catalyst failure has specific attributes. In particular, a heavily deactivated catalyst can still maintain considerable Chabazite structural integrity as determined from X-ray diffraction (XRD), suggesting that structural degradation of the SAPO-34 support alone, due to well-established irreversible hydrolysis^9^, cannot, by itself, explain catalyst failure. Rather, the transformation of SCR active Cu species into inactive ones appears to be a more plausible explanation^7^. However, it is not clear at all how such transformations occur at near-ambient conditions.

Although understanding low-temperature instability of Cu/SAPO-34 is critical for designing more robust SCR catalysts, explicit causes are difficult to identify, because of the intrinsic structural complexity of such catalysts. First, SAPO-34 can be synthesized using a wide variety of Al/P/Si precursors and structure directing agents (SDAs), forming SAPO-34 materials with different T-site distributions and thus, different sensitivities to moisture^9,10^. Second, Cu distributions can be highly complex^6,11–13^. Solution ion exchange, the commonly used method for the synthesis of Cu/SAPO-34 catalysts, inevitably leads to the coexistence of preferred isolated Cu ions and unwanted CuO, as well as uneven Cu distributions within individual SAPO-34 particles^11,12^. This complexity renders Cu transformations under varying hydrothermal treatments difficult to trace and quantify.

To circumvent catalyst complexity described above, a one-pot synthesis approach was applied here to prepare model Cu/SAPO-34 catalysts with uniformly dispersed Cu species that are essentially present as isolated ions at cationic sites (i.e., the SCR active form). To speed up low-temperature hydrolysis of these model catalysts, morpholine was chosen as the primary SDA, which is known to lead to SAPO-34 materials more vulnerable to water attack than with other common SDAs^9^. Atomic Cu dispersion was achieved by using Cu-TEPA (tetraethylenepentamine) complexes as the Cu source, a species that is stable during one-pot synthesis and can also function as a co-SDA^14,15^. By varying the amount of Cu-TEPA, two samples with Cu loadings of 0.71 wt% (Cu1) and 1.90% (Cu2) were synthesized. Details on the compositions of the two catalysts are provided in Table S1. The freshly prepared, dried and calcined samples (designated as “F”) were divided into two parts, one being treated hydrothermally either at 70 °C (designated as “LT”) or 800 °C (“HT”), and second being stored in a typical ambient lab environment (~20 °C, relative humidity ~50%) for 240 days (“S”).

In the main body of the current work, these catalysts are analyzed with a number of spectroscopic and chemical titration methods to elucidate structural changes in SAPO-34 and Cu transformations. The observed structural modifications are then correlated with catalytic performance in the standard NH_3_ SCR reaction (4NO+4NH_3_+O_2_=4N_2_+6H_2_O). In particular, hyperfine sublevel correlation (HYSCORE) spectroscopy, a two-dimensional (2D) pulsed electron paramagnetic resonance (EPR) technique^16^, is used to gain important insight on Cu transformations. The experimental studies are then closely coupled with detailed density functional theory calculation to unravel the underlining destructive mechanism by water which only occurs below 100 °C.

## Results and discussion

### Effects on standard SCR performance

Standard SCR testing was conducted at a few space velocities to measure light-off behaviors and turnover rates under kinetic control^17^. Supplementary Fig. 1a, b presents light-off curves of Cu1 and Cu2 samples in F, LT, and HT forms. For Cu1, both low- and high-temperature hydrothermal treatments induce remarkable deactivation. For Cu2, low (<~200 °C) temperature NO_*x*_ conversions decrease with hydrothermal treatments; yet catalytic performance is maintained (for Cu2-HT) or even enhanced (for Cu2-LT) at higher reaction temperatures. SCR performance of the Cu1-S sample stored under ambient conditions was unexpected. As shown in Supplementary Fig. 2, at reaction temperatures where Cu1-F reaches ~100% NO_*x*_ conversions, Cu1-S only shows NO_*x*_ conversions lower than ~20%. Furthermore, this catalyst becomes less selective in SCR above ~400 °C as indicated by the striking differences between NO_*x*_ and NH_3_ conversions. As will be shown below, however, this sample is much more informative in revealing low-temperature deactivation mechanisms for Cu/SAPO-34 than any other samples studied here. In particular, The Cu2-S sample (not shown), on the other hand, displays essentially identical catalytic performance as Cu2-LT and, as such, was not further explored in this study.

**Fig. 1:**
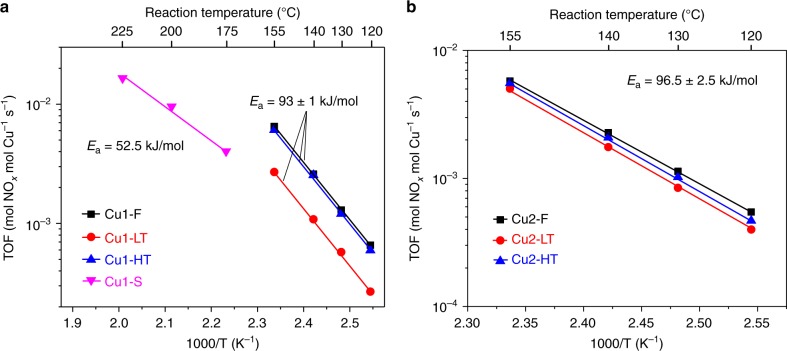
Arrhenius plots for low-temperature standard NH_3_-SCR. **a** Cu1-F/LT/HT catalysts; **b** Cu2-F/LT/HT catalysts. The feed gas contained 360 ppm NO, 360 ppm NH_3_, 14% O_2_, 2.5% H_2_O and balance N_2_. The total gas flow was 1000 sccm, and the gas hourly space velocity (GHSV) was estimated to be ~650,000 h^−1^. TOFs were calculated using EPR active Cu(II) contents in each sample

To obtain more details on the effects of Cu transformations on SCR performance, standard SCR reactivity was also measured under kinetic control (i.e., at low-temperature differential conditions). In this way, turnover frequencies (TOFs) could be calculated assuming that SCR is catalyzed by isolated Cu(II) ions, with the latter quantified using electron paramagnetic resonance (EPR) analysis of hydrated ambient samples (as described below)^17^. Fig. 1a presents TOFs of the Cu1 samples in the form of Arrhenius plots. Cu1-F/LT/HT samples display essentially identical apparent activation energies (93 ± 1 kJ/mol), typical for NH_3_-SCR over Cu/zeolites under kinetic control (i.e., at low conversions in the absence of mass transfer limitations)^17,18^. Similarity in activation energies indicates active sites of the same nature in different samples; however, TOFs for the Cu1-LT sample are ~56% lower than that over Cu1-F, whereas TOFs for Cu1-HT are only ~8% lower than that on Cu1-F. This indicates that a large portion of EPR active isolated Cu(II) species in Cu1-LT are not SCR active, causing the decrease of the apparent TOFs, since these are normalized against total EPR active Cu(II) sites. The Cu1-S sample, in contrast, not only displays substantially lower reactivity even in comparison to Cu1-LT, but also a significantly lower apparent activation energy, suggesting that the nature of active site has been altered. Figure 1b presents the corresponding Arrhenius plots for the Cu2 samples. Again, similar apparent activation energies of 96.5 ± 2.5 kJ/mol are obtained demonstrating kinetic control, and suggesting similar active sites in each of these samples. In comparison to Cu2-F, the Cu2-LT sample experienced larger TOF drop (~26%) than the Cu2-HT sample (~6%). This is consistent with Cu1, where low-temperature hydrothermal aging causes more significant activity loss. It is important to note that the rate losses described above are caused by the water treatments prior to SCR tests. During the short SCR tests, no catalyst deactivation has been observed.

### Textural property analyses and chemical titrations

Multiple standard materials characterizations were performed on the catalysts studied here. In particular, surface area/pore volume and XRD analyses were used to reveal crystallinity changes, temperature-programmed desorption of ammonia (NH_3_-TPD) was applied to probe NH_3_ storage capacity, and temperature-programmed reduction with hydrogen (H_2_-TPR) was used to study Cu ion reduction characteristics. Isolated Cu(II) ion concentrations were measured by double-integrating EPR signals of fully hydrated samples, and quantification was made using solution Cu-imide complexes with known concentrations. Most of the results are collectively shown in Table 1; more detailed descriptions and discussions are presented in Supplementary Figs. 3-6. In brief, these results support the structure-stabilizing role for Cu ions discovered recently^8^, and demonstrate that low- and high-temperature hydrothermal aging have very different effects on the nature of Cu species present in the catalysts. For the Cu1-S sample in particular, in situ XRD during dehydration demonstrates that, while this sample maintains the Chabazite structural integrity in the fully hydrated ambient form, even a very mild thermal treatment causes the structure to collapse indicative of the highly defective nature for this sample (Supplementary Fig. 3b). Next, focus will be given to the transformations of Cu in the Cu1 samples from detailed EPR analyses.

**Table 1:** Textural properties, chemical titration results, and isolated Cu(II) contents of the catalysts

Sample	BET surface area (m^2^/g)^a^	Micropore volume (cm^3^/g)^a^	NH_3_ storage (mmol/g)^b^	H/Cu ratio (100–600 °C)^c^	EPR active Cu (wt%)
Cu1-F	364	0.204	0.825	1.63 ± 0.16	0.70
Cu1-LT	208	0.112	0.577	1.84 ± 0.12	0.67
Cu1-HT	184	0.097	0.314	1.18 ± 0.05	0.60
Cu1-S	86	0.042	0.114	1.98 ± 0.08	0.51
Cu2-F	456	0.241	0.874	1.65 ± 0.04	1.63
Cu2-LT	344	0.189	0.886	1.68 ± 0.09	1.42
Cu2-HT	390	0.210	0.804	1.78 ± 0.08	1.48

^a^Measured with N_2_ adsorption

^b^Measured with NH_3_-TPD

^c^Measured with H_2_-TPR

### Cu transformations **via** 1D and 2D EPR

Supplementary Fig. 7 presents 1D continuous wave (CW) EPR spectra of the hydrated Cu1 samples. In addition to signal amplitude variation, the hyperfine features (enlarged as an insert) also display considerable differences among the samples. From these spectra, g_||_ and A_||_ tensor values were derived and the results are tabulated in Supplementary Table 2. The amounts of EPR active Cu contents were also included in the table. As shown above, the Chabazite structure of the Cu1-S sample collapses during dehydration. Therefore, EPR spectra of the dehydrated Cu1-F and Cu1-S samples were acquired to reveal their differences, and the results are shown in Supplementary Fig. 8. Interestingly, the high-field signal appears to split into two features (apparent g factors g’ = 2.050 and 2.035) for the dehydrated Cu1-S sample. Again, total EPR active Cu contents and g_||_ and A_||_ tensor values were obtained and these are displayed in Supplementary Table 2. Upon structural collapse, even though the majority of Cu species (~56%) remain as isolated Cu(II), the nature of Cu in this sample is very different from other samples as evidenced from H_2_-TPR (Supplementary Fig. 6), and from SCR test (Fig. 1).

**Fig. 2:**
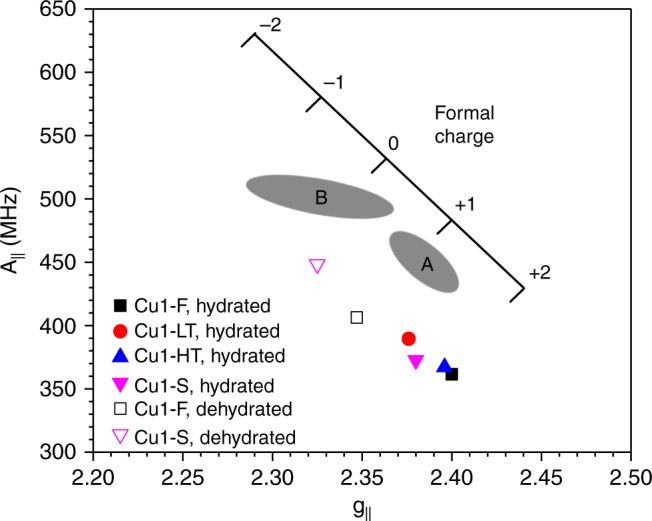
Correlations between (g_||_, **a**_||_) tensor pairs and formal charges of Cu(II) ions in the Cu1 samples. Note that shaded areas “A” and “B” represent typical (g_||_, A_||_) pair positions found in hydrated and dehydrated Cu/zeolite materials, respectively

In a classic study by Carl and Larsen^19^, g_||_ and A_||_ tensors for Cu(II) ions in varied zeolites (in both hydrated and dehydrated forms) were summarized, and a correlation between the formal charge on Cu(II) and these EPR parameters was established. As shown in Fig. 2, g_||_ and A_||_ tensor pairs are typically found in shaded area A for hydrated Cu(II) ions with a formal charge close to +1. Upon dehydration, g_||_ and A_||_ tensor pairs are typically found in shaded area B, i.e., A_||_ increases, whereas g_||_ and formal charge decrease, as a result of stronger Cu(II)-support interactions. For the Cu1 samples studied here, the g_||_ and A_||_ tensor pairs are also plotted in Fig. 2. In comparison to other Cu/zeolites, A_||_ tensor values for our Cu/SAPO-34 samples are considerably lower indicating weaker Cu-support interactions for the latter. In the hydrated form, Cu1-F and Cu1-HT display very similar Cu-support interactions suggesting that high-temperature treatment does not alter the nature of Cu in hydrated samples, whereas both hydrothermal treatment at 70 °C and ambient temperature storage enhance Cu-support interactions, suggesting differences in the nature of Cu sites as opposed to the fresh sample. For the dehydrated Cu1-F and Cu1-S samples, again, the large differences in g_||_ and A_||_ tensor values and “formal” Cu charges indicate the different nature of Cu species in the two samples.

**Fig. 3:**
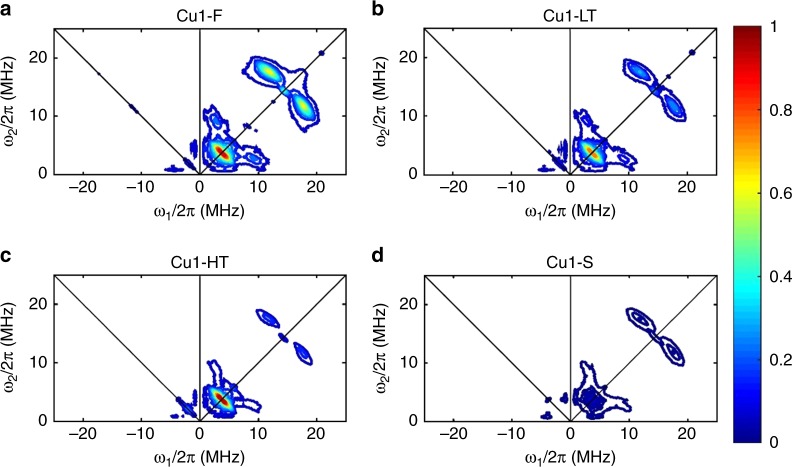
HYSCORE spectra for hydrated Cu1 samples. **a** Cu1-F; **b** Cu1-LT; **c** Cu1-HT; **d** Cu1-S. The four spectra have undergone normalization to signal intensity/videogain/mass, using the same color scale and contour level for the purpose of direct comparison

Next, 2D pulsed EPR (HYSCORE) was applied to further elucidate changes to the local environments of the Cu(II) species in low/high-temperature aging and in room temperature storage. Note that this technique probes interactions between an unpaired electron in Cu(II) and nearby NMR-active nuclei (^27^Al, ^1^H for this system), with an effective range of 0.25–0.8 nm. Figure 3 presents contour spectra for the four hydrated samples normalized with experimental signal intensity/videogain/mass. These spectra are replotted in Supplementary Fig. 9 as surface plots to visually assist spectra comparisons for Fig. 3. Note that each spectrum contains two quadrants. The majority of signals appear in the right (+, +) quadrant, symmetrical along the diagonal. There are some signals that appear in the left (−, +) quadrant, which many times indicates very strong coupling^20^. Note also that signal splitting from the diagonal reflects the strength of coupling between the electron and the nuclei that couples with it: narrow peaks that fall on the diagonals come from nuclei at longer distances; off-diagonal signals originate from strong coupling indicating shorter distances.

For the Cu1-F sample, two groups of features are observed in the (+, +) quadrant: features at lower frequencies are attributed to ^27^Al coupled with Cu(II); features at high frequencies to ^1^H. The strong coupling between Cu(II) and ^1^H indicates that a large portion of isolated Cu(II) sites in this catalyst stays as [Cu(OH)]^+^, one of the two active forms of Cu ions found in the isostructural Cu/SSZ-13 catalysts^3^. For the Cu1-LT catalyst, the ^1^H features appear similar to, but somewhat weaker than Cu1-F, indicating large preservation yet some consumption of Cu–OH bonds. For Cu1-HT, Cu-^1^H coupling becomes much lower in intensity compared with that in Cu1-F and Cu1-LT, indicating substantial consumption of Cu–OH. In comparison to Cu1-F, Cu(II)-^27^Al coupling becomes weaker in the (+, +) quadrant, yet slightly more intensive in the (−, +) quadrant for Cu1-LT. In contrast, Cu(II)-^27^Al coupling for Cu1-HT in both quadrants becomes stronger than Cu1-F. In particular, the near-diagonal signal splits into two features in the (+, +) quadrant, indicating very strong Al–Cu–Al interactions (Fig. 9). HYSCORE simulations (detailed in Supplementary Figs. 10-14) indicate that these strongly coupled ^27^Al nuclei are at a distance of ~2.7 Å, with the features in the (−, +) quadrant that are best simulated with two ^27^Al nuclei rather than one (i.e., with -Al-O-Cu-O-Al- configurations)^21^. The following reaction, i.e., [Cu(OH)]^+^ +H^+^ =Cu^2+^ +H_2_O, best describes high-temperature Cu transformation, which converts thermodynamically less stable [Cu(OH)]^+^ to more stable Cu^2+^ that has been found to occur also in Cu/SSZ-13 during high-temperature hydrothermal aging^18,22^. Note that this transformation does not alter SCR activity since both isolated sites have roughly equal SCR activity (Fig. 1)^23^.

The HYSCORE spectrum of the Cu1-S sample displays a number of surprising features. First, the ^1^H signals are very weak, indicating that the majority of [Cu(OH)]^+^ converts to other species. However, at ambient temperatures and with CHA pores filled with trapped H_2_O, detachment of –OH groups from Cu ions is not readily rationalized. The ^27^Al signals are also an order of magnitude lower than other samples. Note that hydrated Cu(II) ions in CHA cages can couple effectively with framework Al sites (within effective coupling distances of 0.25–0.8 nm). The decreased Cu–Al coupling, therefore, may indicate that a large portion of Cu(II) species are no longer present at cationic exchange positions. In other words, Cu(II) species have migrated a considerable distance from the exchange sites and, therefore, couple few Al sites in the Cu1-S sample.

### ^27^Al, ^29^Si, and ^31^P solid-state NMR

As clearly shown from the EPR analyses, ambient temperature storage induces considerable changes to bonding and locations of Cu ions. Such changes seem likely due, at least in part, to a modified structure of the SAPO-34 zeolite during storage which is not readily captured with XRD (Supplementary Fig. 3). In the following, solid-state NMR was applied to compare Cu1-F and Cu1-S in both hydrated and dehydrated forms. Supplementary Figs. 15a-c present direct polarization (DP) ^27^Al, ^29^Si, and ^31^P spectra for hydrated Cu1-F and Cu1-S samples, together with detailed peak assignments and descriptions. In brief, these spectra collectively demonstrate a much more defective nature for Cu1-S, as evidenced by the higher concentrations of distorted and/or hydrated T sites (even though the Chabazite structure maintains). Supplementary Fig. 16a, b present ^27^Al and ^31^P direct polarization (DP) and cross polarization (CP, with ^1^H) spectra of dehydrated Cu1-F and Cu1-S samples, respectively (in this case, the Chabazite structure collapses for Cu1-S). Again, some descriptions are also presented to further demonstrate the strong coupling between defective Al and H in close vicinity (−OH or strongly bound H_2_O) even after Chabazite structure collapse. In contrast, −OH or strongly bound H_2_O shows much less interaction with P species in the zeolite.

### The nature of Cu–Al interactions below 100 °C

The results shown above, especially EPR and NMR analyses of the Cu1-S and Cu1-LT samples, demonstrate dramatic Cu transformations from the SCR active form (i.e., isolated Cu-ions in cationic positions) to an SCR inactive, but still EPR detectable form (i.e., still as isolated Cu(II)). H_2_-TPR results shown in Supplementary Fig. 6 provide important indications on the nature of the latter Cu moieties. For the Cu1-S sample, reduction centered at ~365 °C is readily assigned to Cu(II) $\rightarrow$ Cu(0) reduction based on the H/Cu quantification. Moreover, the rather high reduction temperature suggests that the Cu moieties in this sample are clearly no longer isolated Cu(II) ions in zeolite cationic positions which are reduced to Cu(I) at much lower temperatures. Rather, the reduction temperature matches very well with reduction of copper aluminate-like species generated during hydrothermal treatment of Cu/zeolites^24^. To elucidate whether Cu-aluminate-like species form in Cu1-S, a physical mixture of CuO and γ-Al_2_O_3_ (containing 1 wt% Cu) was calcined in air at 1000 and 1200 °C, and then subjected to EPR analyses. As shown in Supplementary Fig. 17a, the sample calcined at 1000 °C contains 0.31 wt % EPR active Cu, suggesting formation of CuAl_2_O_4_ with EPR active Cu(II). In contrast, the sample calcined at 1200 °C only contains 0.03 wt% EPR active Cu, suggesting dominance of CuAlO_2_ in which Cu has a +1 oxidation state and is, thus, EPR silent. Importantly, the sample calcined at 1000 °C after dehydration also displays two features in the high field with apparent g factors g’ = 2.050 and 2.031 as shown in Supplementary Fig. 17b. Even though the nature of the two high-field features awaits further elucidation, the similarity between this spectrum and that of the dehydrated Cu1-S sample (Supplementary Fig. 8) strongly suggests formation of Cu-aluminate-like species in the Cu/SAPO-34 sample stored under ambient conditions for nearly a year. In fact, from XRD, HYSCORE and ^29^Si NMR results, strong Cu–Al interactions even occur at ambient temperatures without appreciable destruction of the Chabazite structure; i.e., before extensive desilication of the SAPO-34 substrate occurs. This is believed to be the main reason why a deactivated industrial catalyst still appears “normal” in terms of structural integrity.

Formation of CuAl_2_O_4_-like species requires (1) formation of reactive terminal ≡Al-OH/≡Al(H_2_O)_*n*_ (*n* = 1–3) via hydrolysis of the SAPO-34 substrate^9,25^, and (2) transformation of SCR active Cu(II) ions in cationic positions to Cu moieties that can react with ≡Al-OH/≡Al(H_2_O)_n_ to form CuAl_2_O_4_-like species, or at least its precursors. We have recently shown that [Cu(OH)]^+^ active sites can detach from cationic positions via a hydrolysis chemistry^18,22^, i.e., [Cu(OH)]^+^ + H_2_O = Cu(OH)_2_ + H^+^. This reaction leads to the formation of free Cu(OH)_2_ molecules and regeneration of Brønsted acid sites (≡Si-O(H)-Al≡) that are vulnerable to hydrolysis. Unlike zeolites that dealuminate during hydrothermal treatment, desilication is more facile for SAPO materials^25^. Upon sequential hydrolysis of ≡Si-O(H)-Al≡ bonds, free Si(OH)_4_ and terminal ≡Al-OH/≡Al(H_2_O)_n_ moieties form. In the following, we describe theoretical approaches that were used to gain atomic-level details of these chemistries, particularly why these only occur at near-ambient temperatures (<100 °C).

Here, we simulate how (condensed) water promotes [Cu(OH)]^+^ hydrolysis to Cu(OH)_2_, ≡Si-O(H)-Al≡ hydrolysis, and interactions between Cu(OH)_2_ and terminal Al species. A model Cu/SAPO-34 structure was constructed that contains two hexagonal unit cells (72 T) with Si/P/Al = 1/2/3. The optimized simulation cell parameters are 13.6433 × 23.6310 × 14.8205 Å^3^. One [Cu(OH)]^+^ was used to replace a proton in an 8-membered ring as the reactive site. This structure is simple enough for simulation purposes, and mimics the Cu1 sample composition rather well. As shown in Supplementary Fig. 18, the DFT calculated Cu–Al and Cu-H distances for the optimized structure, 2.86 and 2.37 Å, respectively, are very close to values obtained via HYSCORE simulations (2.74 and 2.48 Å, respectively), thus validating the representativeness of our structural model.

**Fig. 4:**
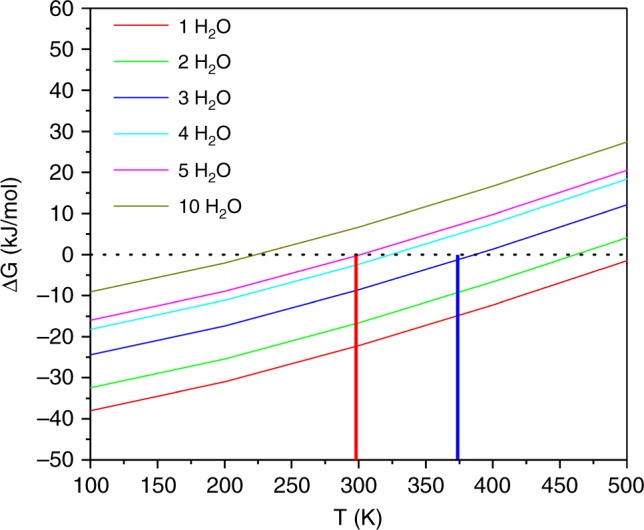
Calculated Gibbs free adsorption energies as a function of temperature. The 6 thin solid curves describe scenarios with different number of H_2_O molecules (1–10) in the vicinity of a Brønsted acid site. The dashed horizontal line represents Gibbs free energy at zero, and the vertical red and blue lines correspond to 298 K and 373 K, respectively

Given the hydrophobic nature of silicoaluminophosphate zeotype materials^11^, water adsorption by Cu/SAPO-34 must be associated with Brønsted acid sites, Cu-ions and other minor defect sites. At low Cu loadings (e.g., Cu1), Brønsted acid sites are considered as the main contributor for H_2_O adsorption. As such, the numbers of water molecules that can be stabilized by Brønsted acid sites as a function of temperature at 1 bar is first simulated. As shown in Supplementary Fig. 19, each Brønsted acid site can accommodate up to ~10 water molecules at 0 K. With increasing temperature or the number of water molecules, neighboring Brønsted acid sites will be involved for their stabilization. Figure 4 presents the calculated Gibbs free adsorption energies^26^ of varying numbers of water molecules in the vicinity of each Brønsted acid site as a function of temperature. It is readily deduced from this simulation that ~3–5 water molecules can be stabilized in the vicinity of each Brønsted acid site at temperatures below 100 °C. From the catalyst compositions shown in Supplementary Table 1, and Si distributions from Supplementary Fig. 15b (note that silica islands do not provide Brønsted acidity^10,27^), it is estimated that each hexagonal unit cell contains ~2–3 Brønsted acid sites. This translates to the availability of ~6–15 water molecules in each unit cell for the hydrolysis chemistries described above. Such estimations can be readily verified experimentally. Desorption of 15 H_2_O molecules from one unit cell corresponds to ~10% weight loss; this value is consistent with typical weight losses of ~10–20% in Cu/SAPO-34 dehydration. Next, it will be shown that the strong solvation effects provided by multiple water molecules and occurring only below 100 °C, play a decisive role in promoting (1) [Cu(OH)]^+^ hydrolysis to free Cu(OH)_2_, (2) ≡Si-O(H)-Al≡ hydrolysis to terminal reactive Al, and (3) Cu(OH)_2_ and terminal Al interactions. At elevated temperatures (e.g., 800 °C), water concentrations within the SAPO-34 cages are negligible, thus precluding such reactions.

**Fig. 5:**
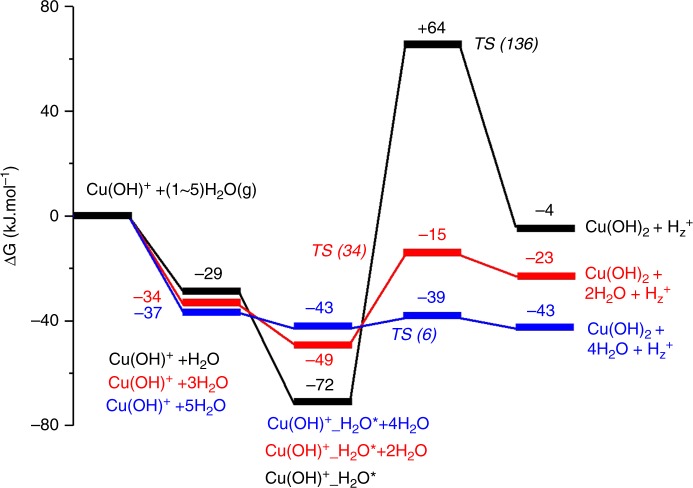
The effects of water concentration on the hydrolysis of the [Cu^II^(OH)]^+^ to Cu(OH)_2_. Optimized structures of each intermediates are given in Supplementary Fig. 20

Figure 5 presents Gibbs free energy diagrams of [Cu(OH)]^+^ cationic site hydrolysis to free Cu(OH)_2_ and H^+^ in the presence of 1, 3, and 5 water molecules. In considerable contrast to a high barrier of 136 kJ/mol when only 1 H_2_O molecule participates in the hydrolysis, the activation barrier for the formation of Cu(OH)_2_ decreases from 34 kJ/mol with 3 water molecules to 6 kJ/mol for 5 water molecules in the vicinity of Cu. The optimized structures of the intermediate states are given in Supplementary Fig. 20. As suggested by previous experimental work^28,29^, the hydrolysis of ≡Si-O(H)-Al≡ and ≡P-O-Al≡ bonds in SAPO-34 leads to formation of terminal Al sites. Our calculations show that the hydrolysis of ≡P-O-Al≡ bonds is highly endothermic under the relevant water concentrations studied here. In contrast, the hydrolysis of ≡Si-O(H)-Al≡ bonds is facile. Previous NMR studies^9,28^ suggest that the hydrolysis chemistry can be depicted as follows: ≡Si-O(H)-Al≡ + 3H_2_O = ≡Si-OH + ≡Al(H_2_O)_3_. As shown in Supplementary Fig. 21, in the presence of 2 extra H_2_O molecules, the activation barrier drops significantly from 69 to 31 kJ/mol, and the entire hydrolysis process also changes from slightly endothermic to exothermic.

Finally, interactions between ≡Al(H_2_O)_3_ and Cu(OH)_2_ (for the formation of a precursor to CuAl_2_O_4_) are simulated. As shown in Supplementary Fig. 22, without the participation of extra water molecules, complexing the two moieties requires a rather high activation barrier of 73 kJ/mol due to repulsion between the two, and the final formation of =Al(µ-O_2_)Cu (a precursor to CuAl_2_O_4_) is endothermic with respect to the initial state. However, when two extra H_2_O molecules are used to solvate Cu(OH)_2_, the activation barrier drops to 15 kJ/mol, and the entire process becomes exothermic. The preceding simulations, intended to be illustrative rather than exhaustive (for example, ≡Al-OH formation and its interactions with Cu(OH)_2_ are not simulated here) because detailed CuAl_2_O_4_ formation pathways are still not clear, do point to the same general conclusion that the presence of multiple water molecules, a more likely scenario at relatively low temperatures, plays a crucial and beneficial role in lowering barriers for the chemistries that lead to the formation of this species.

### Implications to industrial applications

The transportation industry has been puzzled by the slow but irreversible deactivation of Cu/SAPO-34 SCR catalysts for some time in that, despite catalytic performance loss, a deactivated catalyst is otherwise “normal” in comparison to a fresh one as characterized by many standard methods^6,7^. For the Cu2 sample studied here (which has a composition similar to industrial catalysts), indeed, because of its relatively high stability and complexity in Cu identity and distribution, activity loss during low-temperature hydrothermal treatment is difficult to explain. Using our specifically prepared Cu1 sample with uniform Cu distribution and almost singular Cu identity, for the first time, we are able to at least semi-quantitatively follow Cu transformations from SCR active to inactive forms via 1D and 2D EPR. This study is yet another example of utilizing relevant but simplified model catalysts to unambiguously pinpoint key aspects of catalytic processes (in this case deactivation). The most important finding here is that chemistries associated with catalyst deactivation, including structural degradation of the SAPO-34 support and transformation of Cu sites, are greatly facilitated by condensed water within catalyst cages.

Improvements in catalyst design, e.g., by optimizing Si distributions, as well as Cu loadings and distributions, can be used to increase stability of Cu/SAPO-34 catalysts as others have also proposed^15^. In the work by Wang et al.^8^, the authors discovered that Cu/SAPO-34 catalysts free from low-temperature water attack contain saturated amounts of ~2.2 wt% isolated Cu(II) ions as determined via EPR. Such loadings correspond to ~1 isolated Cu(II) ion per hexagonal unit cell. This suggests that: (1) repulsive interactions between mobile water-solvated Cu(II) ions preclude the coexistence of more than one such ions in each unit cell; (2) when each unit cell contains one such isolated Cu(II) ion, it is well protected from low-temperature water attack. Our Cu1-F sample contains an isolated Cu(II) loading of 0.7 wt%, corresponding to one Cu(II) ion in every 4 unit cells, i.e., 3 of 4 unit cells are unprotected from low-temperature water attack. It can be anticipated that unit cells without Cu(II) protection will deform first (not necessarily collapse since the pores are filled with water) from low-temperature water attack, leaving unit cells containing Cu(II) also more vulnerable to water attack. Therefore, the rule that one hexagonal unit cell containing one isolated Cu(II) ion can be used as a guideline for the synthesis of stable Cu/SAPO-34 catalysts. Findings from the current study suggest further that the most important criterion in preventing deactivation of Cu/SAPO-34 catalysts is to prevent the hydrolysis chemistries of ≡Si-O(H)-Al≡ and [Cu(OH)]^+^ sites from occurring at low temperatures. This could be achieved, for example, by flushing the SCR catalyst bed with ammonia, the only SCR relevant molecule that binds more strongly with these sites than H_2_O, thus suppressing H_2_O induced hydrolysis when the catalyst bed temperature decreases to ~100 °C. Our Cu1 catalyst saturated with ammonia indeed shows no sign of deactivation following the same 8-month shelf storage treatment, clearly demonstrating the protective role of ammonia in preventing water damage.

To summarize, using model Cu/SAPO-34 catalysts with homogeneously dispersed and isolated Cu ions, Cu species transformations and SAPO-34 structural degradation were investigated using SCR kinetics, spectroscopy (in particular, EPR and NMR), and DFT simulations. These results for the first time provide a satisfactory explanation for the unusual failure of commercial Cu/SAPO-34 SCR catalysts. Hydrolysis chemistries lead to detachment of isolated Cu ions from CHA cationic positions and concurrent formation of terminal Al sites; reactions between these latter moieties generate SCR inactive Cu-aluminate like species. While optimizing catalyst compositions (Si distribution, Cu loading, etc.) will increase durability of this catalyst, a simple yet practical modification of catalyst pretreatment strategies, e.g. via saturation ammonia adsorption, can effectively prevent the low-temperature hydrolysis of Cu active centers and Brønsted acid sites, providing a useful solution to this important practical problem.

## Methods

### Catalyst preparation and treatment

One-pot Cu/SAPO-34 catalysts presented in this study were synthesized using a method similar to that used by Prakash and Unnikrishnan^30^ for the synthesis of pure SAPO-34, except for Cu addition into the gel for the current case. A Cu-TEPA complex solution, prepared by mixing 5 g of anhydrous CuSO_4_, 6 g of tetraethylenepentamine (TEPA) and 89 g of d.i. water, was used as the Cu source. This complex has been shown to be stable during SAPO-34 synthesis, which also serves as a co-structure directing agent (co-SDA)^15,31^. Morpholine (MOR) was chosen as the primary SDA. Previous studies have shown that this SDA leads to SAPO-34 materials relatively vulnerable to water attack^6,9^. This characteristic is not ideal for applications, but benefited this study of Cu/SAPO-34 degradation. 85% o-phosphoric acid (H_3_PO_4_) was used as the P source; aluminum hydroxide powder (containing ~54% Al_2_O_3_) was used as the Al source, and fumed silica (0.001 μm particle size) was used as the Si source. All chemicals used in our syntheses were purchased from Sigma-Aldrich with purities of analytical grade or better. Synthesis gel molar composition was the following: 0.038 or 0.114 Cu-TEPA: 2 MOR: 0.9 SiO_2_: 0.83 P_2_O_5_: 1 Al_2_O_3_: 60 H_2_O. In a typical synthesis: (1) o-phosphoric acid and d.i. water were first mixed and stirred for 5 min at room temperature. (2) Aluminum hydroxide powder was then slowly added in 5 min under stirring; the slurry was then stirred for 20 min. (3) Fumed silica was added within 10 min and the slurry was stirred for 30 min. (4) Morpholine was added drop by drop under stirring and the slurry was then stirred for 30 min. (5) The Cu-TEPA solution, measured to contain the desired amount of Cu, was slowly added to the slurry under stirring. (6) The suspension was transferred to a 150 ml PTFE lined autoclave containing a magnetic stir bar and sealed. The autoclave was placed above a stir/heat plate and inside a sand bath. Under stirring (500 rpm), the slurry was aged at room temperature for 24 h to facilitate silica hydrolysis. Subsequently, the sand bath temperature was raised to 200 °C in order to start SAPO-34 crystallization. The synthesis was maintained for 48 h under continuous stirring, and then the sand bath was cooled down to room temperature. (7) The solid product was separated with centrifugation, washed twice with d.i. water, and then dried at 120 °C in flowing N_2_ before calcination in static air at 600 °C for 5 h.

The as-prepared catalysts are designated as Cu1-F and Cu2-F, where “F” stands for fresh samples. Cu loadings and Al/P/Si contents of the fresh samples were measured with Inductively Coupled Plasma Atomic Emission Spectroscopy (ICP-AES) conducted at Galbraith Laboratories (Knoxville, TN, USA), with the results displayed in Supplementary Table 1. Low-temperature hydrothermal aging of the fresh catalysts was performed in flowing air containing 10% of water vapor at 70 °C for 48 h. The samples thus generated are named Cu1-LT and Cu2-LT, where “LT” stands for low-temperature aging. High-temperature hydrothermal aging was performed in flowing air containing 10% of water vapor at 800 °C for 16 h. These samples are named Cu1-HT and Cu2-HT, where “HT” stands for high-temperature aging. The fresh samples were also stored in glass vials under typical laboratory environments (~20 °C, relative humidity ~50%) for 240 days. These samples are named Cu1-S and Cu2-S, where “S” stands for stored samples.

### X-Ray diffraction (XRD) measurements

Ex situ powder X-Ray diffraction (XRD) was performed on a PANalytical X’Pert MPD system with a vertical Θ–Θ goniometer (190-mm radius). The X-ray source is a long-fine-focus, ceramic X-ray tube with a Cu anode, operating at 40 kV and 50 mA. The data were collected with 2θ ranging from 5 to 50° using a step size of 0.02°. In situ XRD was performed on a PANalytical X’Pert MPD system with a vertical Θ–Θ goniometer (220 mm radius). The X-ray source was a long-fine-focus, ceramic X-ray tube with a Cu anode, operating at 45 kV and 40 mA. The sample was placed on a sample stage (Anton Paar HTK 1200, temperature range 20–1000 °C) and heated stepwise in dry N_2_ flow from 20 °C to higher temperatures at 20 °C intervals. At each temperature, scanning was conducted from 10 to 50° with a step size of 0.02°.

### Surface area and pore volume measurements

Surface areas (BET method) and micropore volumes (t-plot method) of the samples were measured with a Quantachrome Autosorb-6 analyzer. Prior to measurements, the samples were dehydrated under a vacuum overnight at 250 °C. Note that Cu1-S loses its crystallinity after this treatment.

### Temperature-programmed reduction with H_2_

Temperature-programmed reduction with H_2_ (H_2_-TPR) was performed on a Micromeritics AutoChem II analyzer. After purging the hydrated samples (samples stored in air and saturated with moisture) with pure N_2_ at 10 mL/min at room temperature for 30 min, TPR was carried out in 5% H_2_/Ar at a flow rate of 30 mL/min. Temperature was ramped linearly from ambient to 650 °C at 10 K/min, and H_2_ consumption was monitored with a TCD detector. H_2_ consumption was quantified using Ag_2_O and CuO standards.

### NH_3_ temperature-programmed desorption

NH_3_ temperature-programmed desorption (NH_3_-TPD) was used to measure NH_3_ adsorption on Lewis and Brønsted acid sites in the catalysts. NH_3_-TPD was carried out using our SCR reaction system, with NH_3_ detection via an online MKS 2030 FTIR analyzer. A total of 60 mg of catalyst (60–80 mesh) was used for each measurement, and the following experimental steps were followed: (1) heat the sample to 550 °C in O_2_/N_2_ (300 sccm, 14% O_2_) and keep at 550 °C for 30 min; (2) stop O_2_ flow, maintain N_2_ flow, and cool sample to NH_3_ adsorption temperatures of 100 °C; (3) adsorb NH_3_ (500 ppm in N_2_) until outlet NH_3_ concentrations remain constant for 1 h; (4) turn off the NH_3_ flow and purge with N_2_ for 1 h at the adsorption temperature; and (5) while measuring NH_3_ concentrations in the outlet, ramp from the adsorption temperature to 600 °C at 10 °C /min, and maintain at 600 °C until NH_3_ desorption becomes undetectable.

### Electron paramagnetic resonance (EPR) measurements

Electron paramagnetic resonance (EPR) experiments were carried out on a Bruker E580 X-band spectrometer. Powder samples (∼15 mg) were contained in 4 mm OD quartz tubes. For CW experiments it is equipped with a SHQE resonator and a continuous flow cryostat. During spectral acquisition, microwave power was 200 mW, and the frequency was 9.86 GHz. The field was swept 1500G in 84 s and modulated at 100 kHz with a 5G amplitude. A time constant of 20 ms was used. The isolated Cu(II) contents were quantified using the following method. Typically, spectra acquired at 125 K were first double-integrated to obtain signal areas, which are proportional to EPR active isolated Cu(II) contents. To quantify these, a series of standard solutions with different isolated Cu(II) concentrations were prepared by dissolving Cu(NO_3_)_2_·2.5H_2_O and imidazole (Sigma-Aldrich, 99%) in ethylene glycol (Sigma-Aldrich, 99.8%). Imidazole was used here to coordinate with Cu(II) ions to prevent formation of EPR silent Cu dimers. The linear calibration curve generated using the integral areas and Cu contents of the standard solutions was then used for quantification of isolated Cu(II) in the catalysts. For pulsed EPR experiments the spectrometer was outfitted with an MD-5 dielectric resonator and an Oxford liquid helium continuous flow cryostat which maintained the temperature at 10 K. Typical parameters for HYSCORE experiments with a pi/2-tau-pi/2-t1-pi-t2-pi/2 sequence were: pi/2 = 8 ns, pi = 16 ns, tau = 128 ns and t1, t2 ranging from 200 ns to 2248 ns in 128 steps of 16 ns. The recycle delay was ~ 500 us and typically 1024 shots were collected for each delay.

### Nuclear magnetic resonance (NMR) measurements

Nuclear magnetic resonance (NMR) experiments were performed on powder samples either fully hydrated or after evacuation at 10^−3^ Torr and 150 °C for 12 h (dehydrated). Note again that this dehydration treatment causes Cu1-S to lose its crystallinity. All experiments were conducted under a dry N_2_ atmosphere. ^27^Al and ^31^P direct polarization (DP) experiments were carried out on a Varian VNMRS system operating at 14.1 T utilizing a 4 mm TR probe operating in DR mode. Direct polarization experiments with ^1^H decoupling were conducted utilizing a ^1^H frequency of 599.8517, continuous wave decoupling fields of 45 kHz for both ^27^Al and ^31^P, and ^27^Al and ^31^P frequencies of 156.3056 and 242.8129, respectively. Calibrated π/20 pulses of 0.50 us, spinning frequency of 15 kHz, a 50 kHz spectral window, 2048 complex points, and a 2 s pulse delay were utilized to acquire 512 time-averaged scans for ^27^Al DP experiments. Time domain free induction decays were apodized with exponential functions corresponding to 200 Hz of Lorentzian line broadening prior to Fourier transformation. A π/2 pulse of 5.0 us, a spinning frequency of 15 kHz, 100 kHz spectral window, 4096 complex points, and a 240 s pulse delay were utilized to acquire 4 time-averaged scans for ^31^P DP experiments. Time domain free induction decays were apodized with exponential functions corresponding to 100 Hz of Lorentzian line broadening prior to Fourier transformation. ^29^Si DP experiments were carried out on a Varian VNMRS system operating at 20T utilizing a 4 mm TR probe operating in DR mode. Direct polarization experiments with ^1^H decoupling were conducted utilizing a ^1^H frequency of 849.7299 and a continuous wave decoupling field of 50 kHz. A total of 640 time-averaged transients were acquired utilizing a π/2 pulse of 6.0 μs at a ^29^Si frequency of 168.8018, spinning frequency of 10 kHz, a 50 kHz spectral window, 5024 complex points, and a 120 s pulse delay. Time domain free induction decays were apodized with exponential functions corresponding to 300 Hz of Lorentzian line broadening.

### SCR reaction tests

NH_3_-SCR reaction was measured using a plug flow reaction system described elsewhere^17^. Powder samples were pressed, crushed and sieved (60–80 mesh) prior to use. For standard SCR, the feed gas contained 360 ppm NO, 360 ppm NH_3_, 14% O_2_, 2.5% H_2_O and balance N_2_. H_2_O was introduced to the feed by passing balance gas N_2_ through a water saturator. All of the gas lines were maintained at ~120 °C to avoid water condensation. The total gas flow was 1000 sccm. For “light-off” experiments, 200 mg catalyst was used, and the gas hourly space velocity (GHSV) was estimated to be ~200,000 h^−1^. For low-temperature kinetic studies, NO_*x*_ and NH_3_ conversions were maintained low (<15%). In this case, 60 mg catalyst was used, resulting in a GHSV of ~650, 000 h^−1^. Concentrations of reactants and products were measured by an online MKS 2030 FTIR analyzer. Prior to reaction testing, the catalysts were first pretreated in a 14% O_2_/N_2_ flow for 1 h at 500 °C. The following equations were used to calculate NO_*x*_ and NH_3_ conversions:

1 $${\rm NO}x\,{\rm conversion}{\mathrm{\% }} = \frac{{({\rm NO} + {\rm NO}_2)_{{\rm inlet}} - ({\rm NO} + {\rm NO}_2 + {\rm N}_2{\rm O})_{{\rm outlet}}}}{{({\rm NO} + {\rm NO}_2)_{{\rm inlet}}}} \times 100,$$

2 $${\rm NH}_3\,{\rm conversion}{\mathrm{\% }} = \frac{{({\rm NH}_3)_{{\rm inlet}} - ({\rm NH}_3)_{{\rm outlet}}}}{{({\rm NH}_3)_{{\rm inlet}}}} \times 100.$$

### Theoretical calculations with DFT

All periodic DFT calculations were carried out employing a mixed Gaussian and plane wave basis sets implemented in the CP2K code^32^. Core electrons were represented with norm-conserving Goedecker-Teter-Hutter pseudopotentials^33–35^, and the valence electron wavefunction was expanded in a triple-zeta basis set with polarization functions^36^ along with an auxiliary plane wave basis set with an energy cutoff of 400 Ry. The generalized gradient approximation exchange-correlation functional of Perdew, Burke, and Enzerhof (PBE)^37^ was used. The adsorption and reaction intermediate configurations were optimized with the Broyden-Fletcher-Goldfarb-Shanno (BGFS) algorithm with SCF convergence criteria of 1.0 × 10^−8^ au. The calculated total energy difference was negligible (<0.01 eV) when the maximum force convergence criteria of 0.001 hartree/bohr was used. The short-range van der Waals dispersion interaction was compensated using semi-empirical DFT-D3 scheme^38^.

The SAPO-34 zeolite structure was modeled using two hexagonal unit cell with the size parameters of 13.6433 × 23.6310 × 14.8205 Å^3^. To mimic experimental Cu1 composition (Cu_0.34_Al_17.4_P_11.7_Si_6.9_O_72_), the simulated system shown in Supplementary Fig. 18 was constructed with a composition of CuAl_36_P_24_Si_12_H_12_O_145_ where the Si/Al/P ratio was 1:3:2 and one proton (H^+^) at a Brønsted acidic site (BAS) was replaced by a [Cu^II^(OH)]^+^, which was located in the window of eight membered ring (8MR) of SAPO-34 zeolite^23,39–41^. It has been well-established that confinement and steric hindrance can strongly affect the stabilities of adsorption states and reaction intermediates in the zeolites^42–46^, As such, Gibbs free energy changes (ΔG) that account for the important entropic contribution (ΔS) and zero-point energy (ZPE) corrections were calculated using statistical thermodynamic method, with details provided elsewhere^47^. Transition states of elementary reaction steps were located using the climbing image nudged elastic band (CI-NEB) method^48,49^ with seven intermediate images along the reaction pathway between initial and final states. The identified transition states were confirmed by a vibrational analysis.

## Supplementary information

Supplementary Information

Peer Review

## Data Availability

All data supporting this study and its findings are available within the article and its Supplementary Information or from the corresponding author upon reasonable request.
