# Supplementary material for: Unraveling the mysterious failure of Cu/SAPO-34 selective catalytic reduction catalysts
Source: Nat Commun. 2019 Mar 8;10:1137. doi: 10.1038/s41467-019-09021-3 (PMC6408507; doi:10.1038/s41467-019-09021-3)
Supplement: Supplementary file 1 — Supplementary Information [file 41467_2019_9021_MOESM1_ESM.pdf]

Supplementary Information

Unraveling the mysterious failure of Cu/SAPO-34 selective catalytic reduction catalysts

Wang, A. et al.

Supplementary Table 1. Elemental analysis results from ICP, and estimated unit cell compositions of the two model Cu/SAPO-34 catalysts.

Sample	Cu Content (wt.%)	Al content (wt.%)	P content (wt.%)	Si content (wt.%)	Estimated hexagonal unit cell stoichiometry
Cu/SAPO-34 (1)	0.71	15.4	11.9	6.37	$Cu_{0.34}Al_{17.4}P_{11.7}Si_{6.9}O_{72}$
Cu/SAPO-34 (2)	1.90	15.5	12.5	5.74	$Cu_{0.91}Al_{17.5}P_{12.3}Si_{6.2}O_{72}$

In estimating hexagonal unit cell (36 T and 72 O) stoichiometry, it is assumed that all Al/P/Si are in tetrahedral framework positions. For the Cu1 sample, approximately 1 Cu atom is found in every 3 unit cells. For the Cu2 sample, roughly 1 Cu atom is found in each unit cell. In estimating Brønsted acid sites in these samples, it is worthwhile noting that only isolated Si sites generate Brønsted acidity and paired Si to larger silica islands do not.[1-4] Using the ^{29}Si NMR results shown in Supplementary Fig. 16b for Cu1 (isolated Si appears at ~-90 ppm and silica islands appear at ~-111 ppm), it can be estimated that each unit cell contains 2-3 Brønsted acid sites. The ^{29}Si NMR spectrum for Cu2 is not shown but appears very similar to that of Cu1 suggesting similar numbers of Brønsted acid sites. A $[Cu(OH)]^+$ SCR active species replaces one H^+ while an active Cu^{2+} replaces two H^+.

Supplementary Table 2. EPR active Cu contents (wt%) in various Cu1 samples and the simulated $A_{||}$ and $g_{||}$ tensor values.

Sample	EPR active Cu (wt.%)	$A_{\|\|}$	$g_{\|\|}$
Cu1-F (hydrated)	0.70	129	2.400
Cu1-LT (hydrated)	0.67	139	2.376
Cu1-HT (hydrated)	0.60	131	2.396
Cu1-S (hydrated)	0.51	133	2.380
Cu1-F (dehydrated)	0.54	145	2.347
Cu1-S (dehydrated)	0.39	160	2.325

EPR active Cu (i.e., isolated Cu(II) ion) contents were obtained by double-integrating EPR spectra of hydrated and dehydrated samples measured at 125 K, and quantified against Cu-imide standard Cu solutions with known concentrations.[5,6] For hydrated Cu1-F/LT/HT/S samples, these values were used to calculate turnover frequencies shown in Fig. 1 of the main text. It is important to note that hydrothermal treatments and ambient temperature storage have rather minor effects on the EPR detectability of Cu(II) species, indicating rather minor Cu(II) agglomeration (which will render EPR silence) during these treatments. Dehydration causes some loss of EPR active Cu, attributed to Cu(II) agglomeration and/or Cu(II) autoreduction to Cu(I).[7] Note in particular that the Cu1-S sample still contains ~60% of isolated Cu(II) sites after dehydration despite complete Chabazite structure collapse (Supplementary Fig. 3b). Variations of the $A_{||}$ and $g_{||}$ tensors are discussed in more detail in the main text.

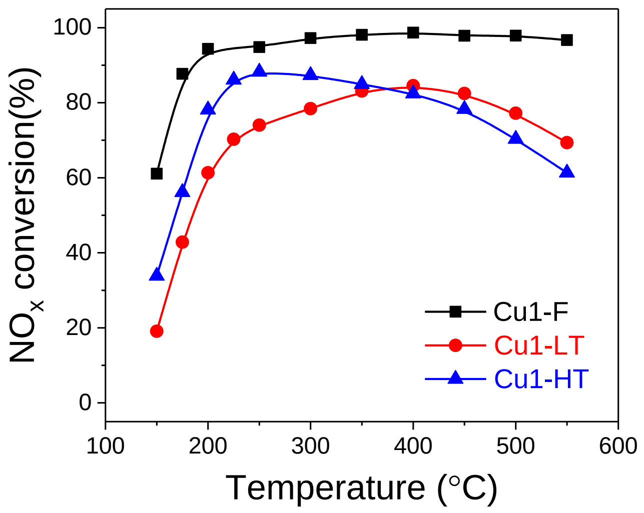

a

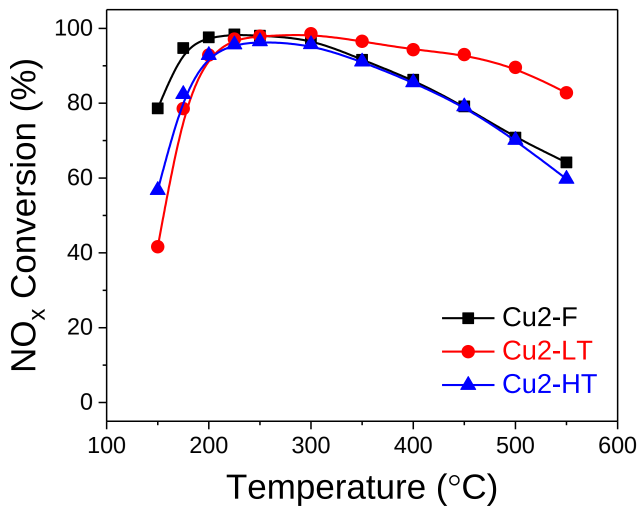

b

Supplementary Fig. 1 NO_x conversion versus temperature curves in standard SCR. **a** Cu1-F/LT/HT; **b** Cu2-F/LT/HT samples. The feed gas contained 360 ppm NO, 360 ppm NH_3, 14% O_2, 2.5% H_2O and balance N_2. The total gas flow was 1000 sccm, and the gas hourly space velocity (GHSV) was calculated to be ~200,000 h^{-1} for a catalyst amount of 200 mg.

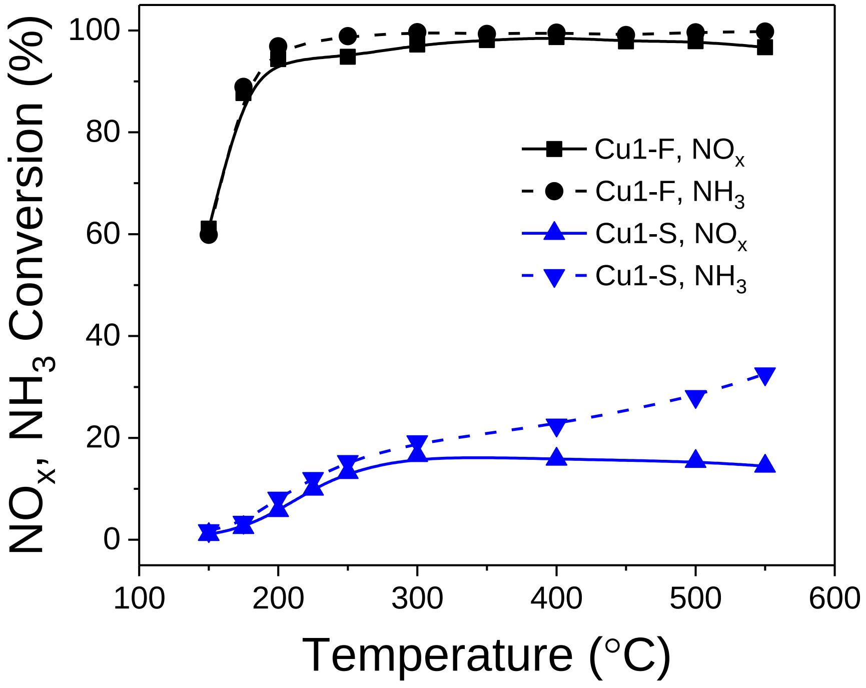

Supplementary Fig. 2 NO_x and NH_3 conversions versus temperature curves for Cu1-F and Cu1-S samples in standard SCR. The feed gas contained 360 ppm NO, 360 ppm NH_3, 14% O_2, 2.5% H_2O and balance N_2. The total gas flow was 1000 sccm, and the gas hourly space velocity (GHSV) was calculated to be ~200,000 h^{-1} for a catalyst amount of 200 mg.

Note that Cu1-F is much more active than Cu1-S, reaching ~100% NO_x conversions at temperatures ≥ 200 °C. In contrast, the Cu1-S sample is much less active, reaching NO_x conversions no higher than 20% at any reaction temperature. Note also that the Cu1-F sample is highly selective in SCR as evidenced by the similar NO_x and NH_3 conversions at each reaction temperature. However, the Cu1-S sample becomes less selective in SCR at reaction temperatures above ~400 °C due to the presence of Cu moieties that are active in catalyzing NH_3 oxidation ($4NH_3 + 3O_2 = 2N_2 + 6H_2O$) at such elevated temperatures.

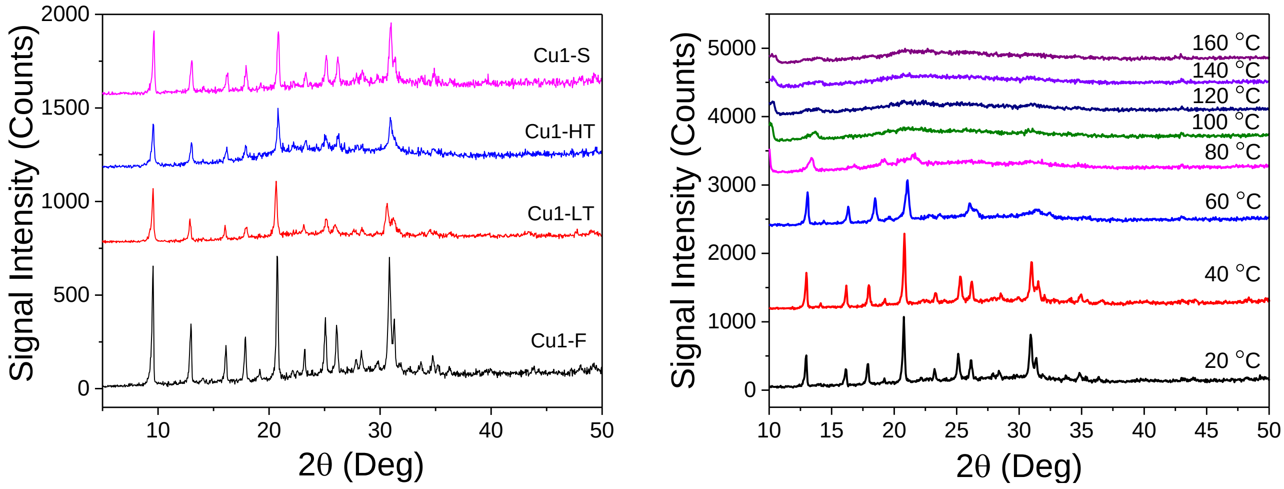

a **b**

Supplementary Fig. 3 XRD patterns for the Cu1 samples. **a** hydrated Cu1 F/LT/HT/S samples; **b** Cu1-S sample during in situ dehydration.

As shown in Supplementary Fig. 3a, diffraction peak intensities for the low- and high-temperature treated and ambient temperature stored samples all decrease substantially in comparison to the fresh catalyst, suggesting partial loss in crystallinity. This is consistent with surface area and pore volume analysis results shown in Supplementary Table 1. From XRD alone, the hydrated Cu1-S sample does not appear to degrade more than Cu1-LT/HT samples. However, this sample does show lower surface area/pore volume in comparison to the Cu1-LT/HT samples (Table 1, main text). Moreover, Cu1-S displays the most dramatic degradation in terms of SCR performance (Supplementary Figs. 1, 2). From in situ XRD measurements during Cu1-S dehydration displayed in Supplementary Fig. 3b, this discrepancy is readily rationalized. The stored, hydrated Cu1-S sample, even though undergoing extensive ≡Si-O(H)-Al≡ hydrolysis during storage, still maintains the Chabazite structure due to space filling of trapped H_2O in pores. Upon H_2O removal at temperatures approaching 100 °C, the CHA structure collapses irreversibly. Importantly, as discussed in the main text, it is not CHA structural collapse, but rather Cu transformation prior to that, that is ultimately responsible for catalyst deactivation. This is why some industrial Cu/SAPO-34 catalysts fully deactivate but still maintain considerable CHA structural integrity.

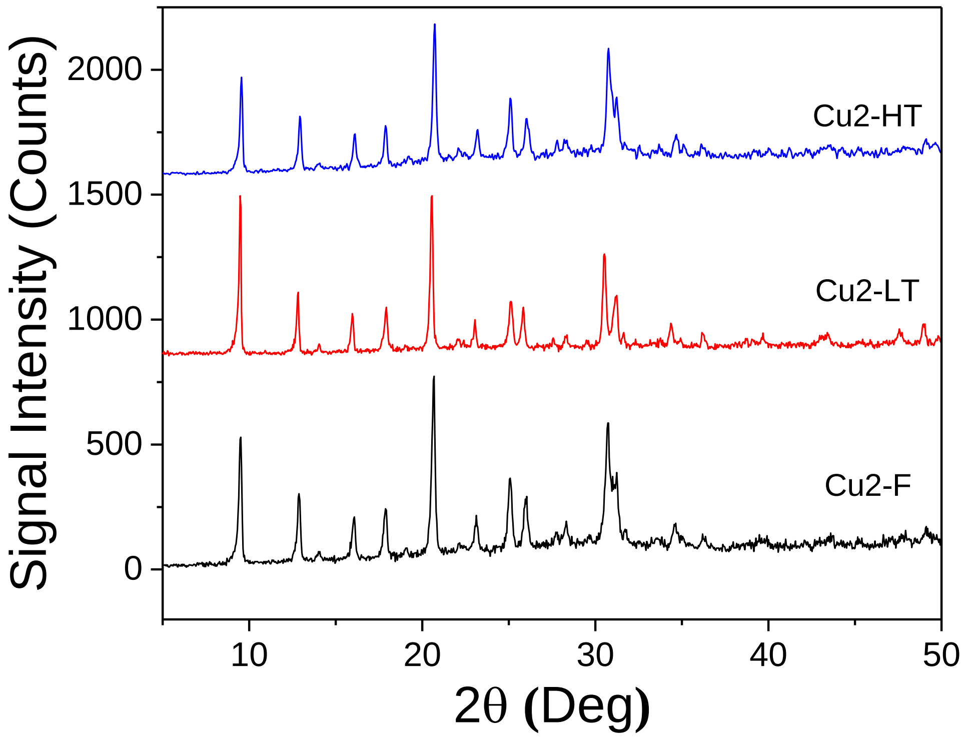

Supplementary Fig. 4 XRD patterns for the hydrated Cu2 samples.

In contrast to the Cu1 samples (Supplementary Fig. 3a), the Cu2-LT/HT samples display XRD patterns closer to Cu2-F, indicating much less structural degradation. Again, this is consistent with surface area/pore volume analysis results shown in Table 1 of the main text. This result is consistent with recent literature findings by Wang et al.,[8] who discovered that Cu can stabilize the SAPO-34 structure. However, as discussed in the main text, increasing Cu content complicates Cu distribution and identity, thus precluding ready elucidation of catalyst deactivation mechanisms for these samples.

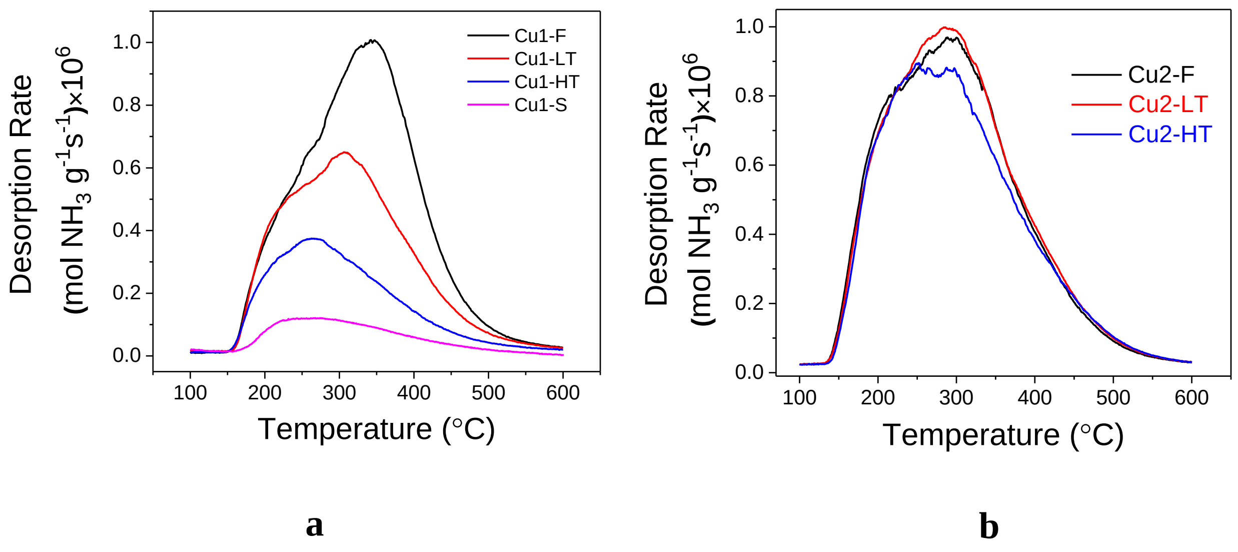

Supplementary Fig. 5 NH_3-TPD results for Cu1 and Cu2 samples. **a** Cu1-F/LT/HT/S; **b** Cu2-F/LT/HT.

NH_3-TPD was applied to investigate NH_3 storage capacities of the samples. NH_3 adsorption and purging with dry N_2 was carried out at 100 °C. NH_3 desorption yields are presented in Table 1 of the main text. The NH_3 desorption states are not well resolved but, based on prior literature, desorption below ~300 °C can be attributed to desorption from Cu and other weakly acidic sites (e.g., extraframework Al). Desorption above ~300 °C is readily assigned to desorption from strong Brønsted acid sites (i.e., ≡Si-O(H)-Al≡).[9-11] For the Cu1 samples, two effects are notable following low- and high-temperature hydrothermal aging: total NH_3 storage capacity loss and Brønsted acidity loss, in line with the rather dramatic irreversible hydrolysis of ≡Si-O(H)-Al≡ sites during these treatments. For the Cu1-S sample, the extremely low NH_3 storage capacity is due also to the CHA structure collapse during the pretreatment (Supplementary Fig. 3b). In contrast, the Cu2 samples show little difference in NH_3 storage capacities, consistent with the much improved hydrothermal stability as the Cu loading increases. It is important to note, however, a strong correlation between NH_3 storage capacity and catalytic activity for individual active sites cannot be established. For example, even though Cu1-HT displays much lower NH_3 storage than Cu1-F, low-temperature TOFs for these two catalysts are rather similar (Fig. 1a).

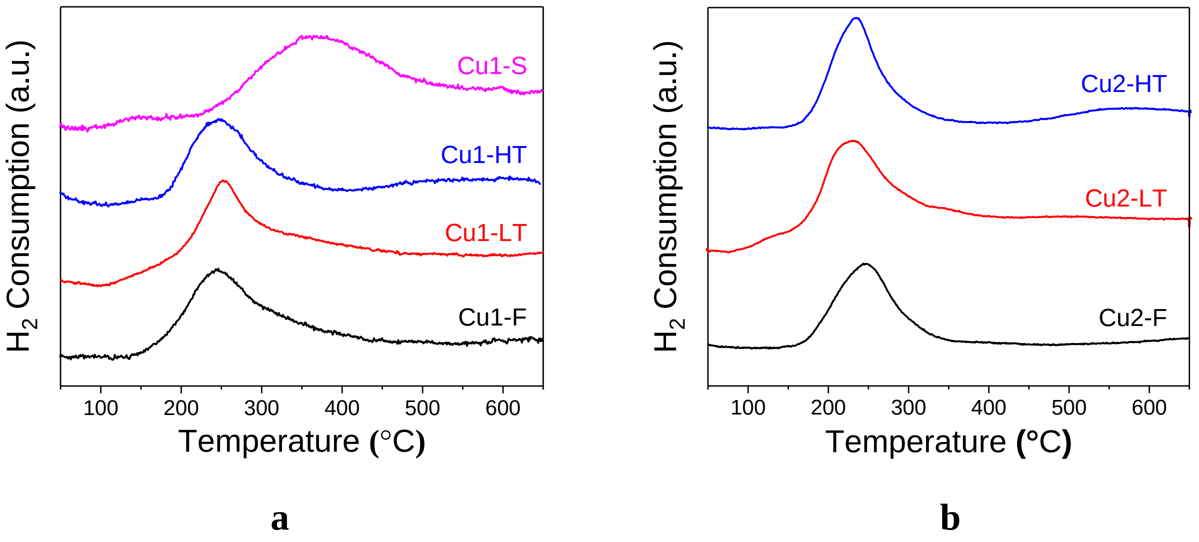

Supplementary Fig. 6 H_2-TPR results Cu1 and Cu2 samples. **a** Cu1-F/LT/HT/S; **b** Cu2-F/LT/HT.

According to previous studies,[5,12] isolated Cu(II) ions are sequentially reduced via the following two-step reactions:

$$Cu^{2+} + ½ H_2 = Cu^{+} + H^{+} \text{ or } [Cu(OH)]^{+} + ½ H_2 = Cu^{+} + H_2O \quad (1)$$

$$Cu^{+} + ½ H_2 = Cu^{0} + H^{+} \quad (2)$$

CuO clusters/particles, on the other hand, are reduced to Cu^0 in a single step:

$$CuO + H_2 = Cu^{0} + H_2O \quad (3)$$

We have shown before that a high-temperature sample pretreatment in inert atmospheres can cause "autoreduction" of some Cu(II) sites (i.e., Cu(II) → Cu(I)).[12] To avoid this chemistry, the hydrated samples were not treated at elevated temperatures prior to TPR. Next, H_2 consumption peaks between 100 and 600 °C are integrated and quantified using CuO reduction as a calibration, and the results are shown as follows (presented as H/Cu ratio):

Supplementary Table 3 H_2 consumption (presented as H/Cu ratio) between 100 and 600 °C during TPR.

Sample	Cu1-F	Cu1-LT	Cu1-HT	Cu1-S	Cu2-F	Cu2-LT	Cu2-HT
H/Cu ratio	1.63±0.16	1.84±0.12	1.18±0.05	1.98±0.08	1.65±0.04	1.68±0.09	1.78±0.08

The quantification results shown in Supplementary Table 3 above indicate that some Cu(II) ions in Cu1-F/LT/HT are only reduced to Cu(I) below 600 °C (i.e., H/Cu < 2). For Cu1-HT in particular,

the majority of Cu(II) are only reduced to Cu(I) below 600 °C (H/Cu = 1.18±0.05). This is consistent with the fact that most of the Cu(II) ions migrate to the energetically most stable cationic positions during high temperature hydrothermal aging, and become more difficult to reduce to Cu^0. On the other hand, the vast majority, if not all of the Cu(II) sites in Cu1-S are reduced to Cu^0 below 600 °C (H/Cu = 1.98±0.08). It is also interesting to note that reduction peaks for Cu1-F/LT/HT samples are centered at ~250 °C (Supplementary Fig. 6a), attributed to reduction of isolated Cu ions in cationic positions. The same assignment can be made to the Cu2-F/LT/HT samples (Supplementary Fig. 6b). However, reduction peak for Cu1-S is centered at ~370 °C, clearly due to changes in the nature of the Cu species; that is, formation of Cu-aluminate-like species. Note that the reduction temperature of ~370 °C found here is very consistent with reduction temperatures of Cu-aluminate-like species formed during hydrothermal aging of Cu/zeolites.[13] Based on EPR measurements shown in the main text, the reduction can be described using the following equation:

$$CuAl_2O_4 + H_2 = Cu^0 + H_2O + Al_2O_3 \quad (4)$$

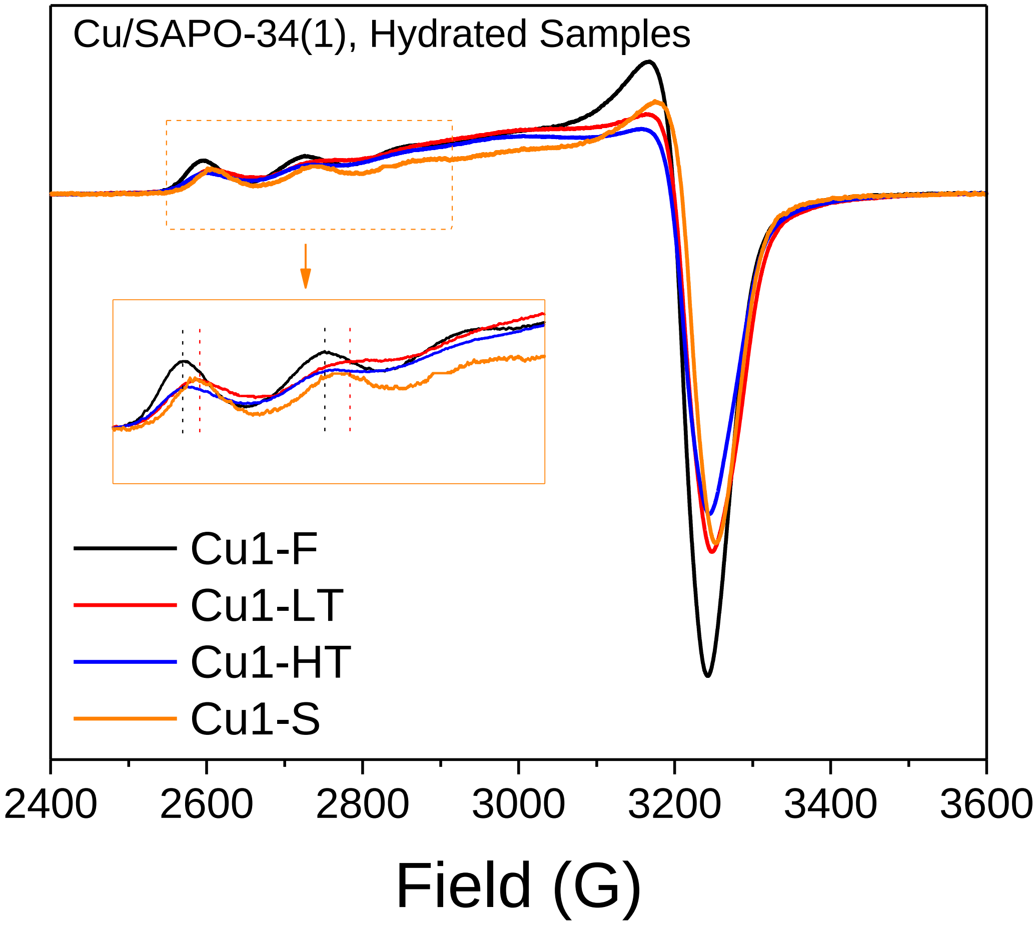

Supplementary Fig. 7 Continuous wave (CW) EPR spectra of the hydrated Cu1 samples. Measurements were conducted at 125 K.

Note that in the high field, all samples display single features at ~3250 G attributed to hydrated, isolated Cu(II) ions.[14] In the hyperfine region, each type of Cu(II) species displays 4 equally-spaced hyperfine features as a result of the S = 1/2 ground state coupling to the I = 3/2 nuclear spin of Cu, splitting spectral features into 2I + 1 = 4 lines;[15] as shown in the insert, features in the lower fields are better resolved. From the peak positions, it can be qualitatively suggested that Cu(II) ions in Cu1-F and Cu1-HT are alike, and Cu(II) ions in Cu1-LT and Cu1-S are similar. More detailed (g, A) tensor analyses are provided in the main text.

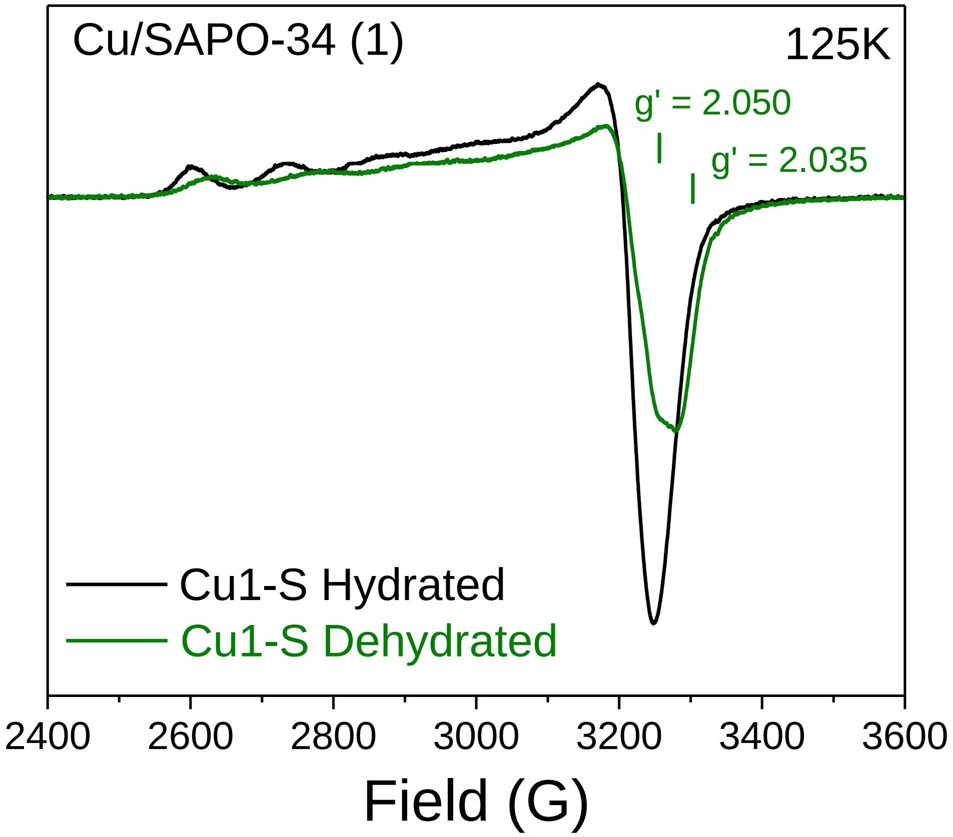

Supplementary Fig. 8 Comparison between continuous wave (CW) EPR spectra of hydrated and dehydrated Cu1-S samples. Measurements were conducted at 125 K.

As shown in Supplementary Fig. 3b, the CHA structure for the Cu1-S sample becomes completely damaged upon dehydration. However, the amounts of EPR active Cu(II) only experienced a moderate decrease from 0.51 wt% to 0.39 wt% (Supplementary Table 2). Note that the EPR active Cu(II) moieties in the dehydrated sample no longer occupy cationic positions as evidenced from their greatly altered $g_{||}$ and $A_{||}$ tensor values as compared to Cu1-F; however, they must still be isolated to be EPR active. Importantly, these species display two features at high field with apparent g tensor values of g’ = 2.050 and 2.035. As will be shown later, this allows us to suggest their chemical nature using model compounds.

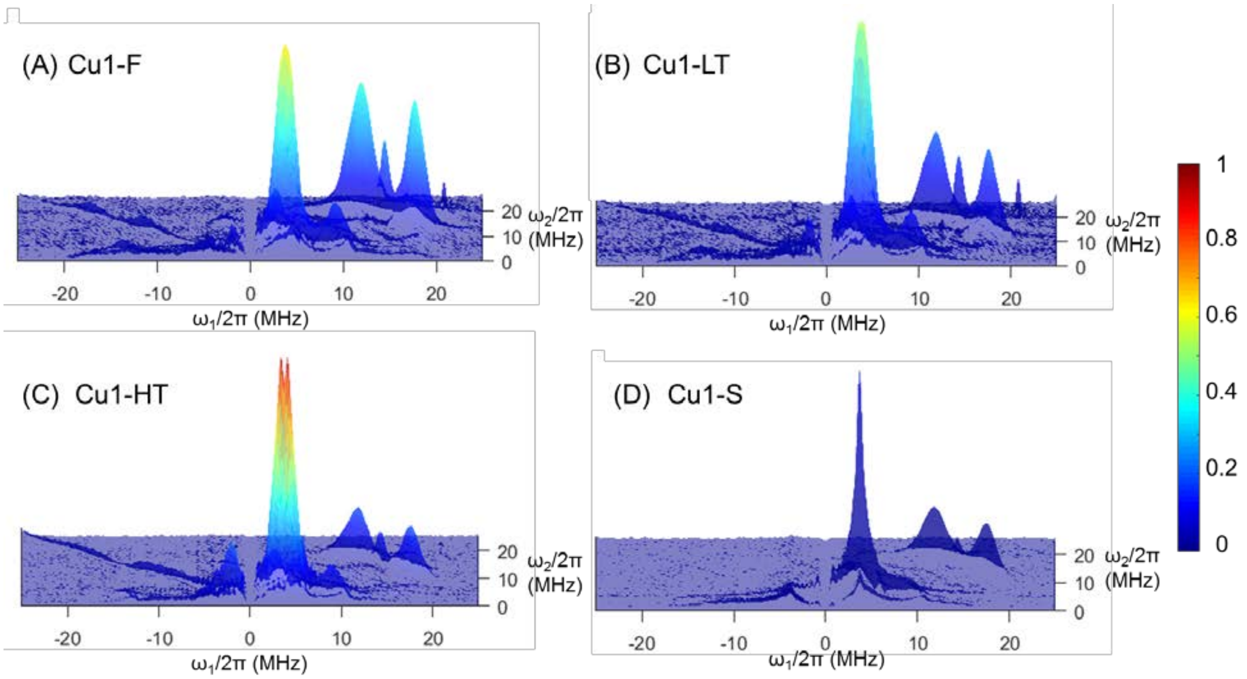

Supplementary Fig. 9 Surface plots of HYSCORE experimental spectra for hydrated Cu1 samples. **A** Cu1-F; **B** Cu1-LT; **C** Cu1-HT; **D** Cu1-S. All spectra are normalized by experimental signal intensity/videogain/mass; the Cu1-S spectrum has also been "symmetrized".

These spectra, in combination with the contour plots shown in Fig. 3 of the main text, allow detailed semi-quantitative comparisons between samples (described in the main text).

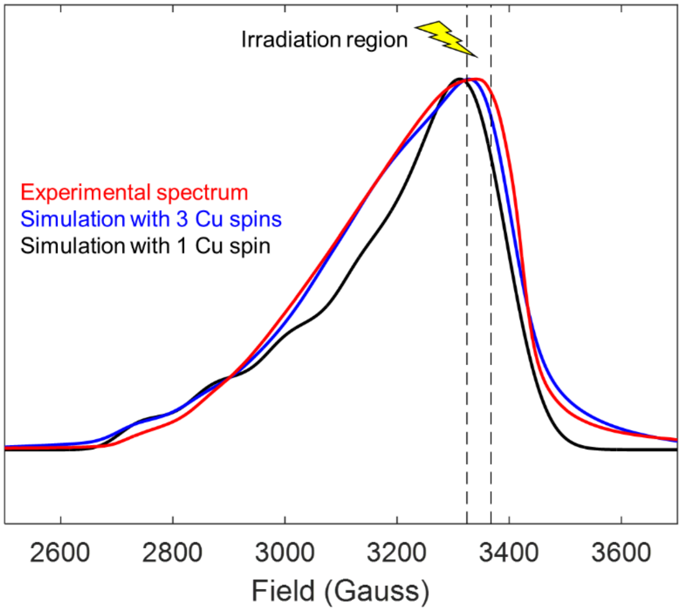

Supplementary Fig. 10 Experimental field sweep spectrum of hydrated Cu1-HT (red) compared to simulation spectra using 3 copper centers (blue) and 1 copper center (black).

The HYSCORE simulations were performed based on the “saffron” function in EasySpin, a Matlab package for spectral simulation and analysis in EPR.[16] First, an experimental HYSCORE spectrum was separated into ^{1}H and ^{27}Al regions and normalized individually. The experimental parameters, such as magnetic field, excitation frequency, number of points, dwell time, pulse width, and pulse delay, were copied directly from the HYSCORE experiments, while axial **g** tensor values and hyperfine parameters of Cu were obtained using the least-squares fitting program, “esfit”, in EasySpin by fitting the experimental solid-state CW EPR spectra. Note that the irradiation was in the $g_{\perp}$ region of the spectrum with an excitation bandwidth of 125 MHz, as shown in Supplementary Fig. 10.

When simulating the ^{1}H region, the isotropic hyperfine parameter, A_{iso}, was varied between -12 and 12 MHz with an interval of 0.4 MHz, the axial hyperfine parameter $T_{\perp}$ was varied between 0 and 12 MHz with an interval of 0.2 MHz, while the orientation angle, θ_{I}, the angle between the z

axis direction ($g_{\|}$) and the vector that connects the electron spin and the nucleus, was set at 0°, 45° and 90°. Accordingly, a total of 3×60×60 ^{1}H HYSCORE spectra were simulated. To facilitate comparison between experimental and simulated spectra, diagonal peaks resulting from coupling with distant protons and diagonal noises in the experimental ^{1}H spectra were manually removed. A nonnegative linear least-squares function ("lsqnonneg") in Matlab was used to obtain a normalization factor, n, for each simulation spectrum to best fit the experimental spectrum, then the sum of the square of the difference between every single point in the experimental spectrum and the corresponding point in a normalized simulation spectrum was calculated and plotted as a function of θ_I, A_{iso}, and $T_\perp$ according to the following equation (Supplementary Fig. 11):

$$\Delta\,(\theta_I,\,A_{iso},\,T_\perp) \;=\; \sum_{i=1}^{N\times N}(S\mathrm{exp}_i - n\times S\mathrm{sim}(\theta_I,\,A_{iso},\,T_\perp)_i)^2 \qquad (5)$$

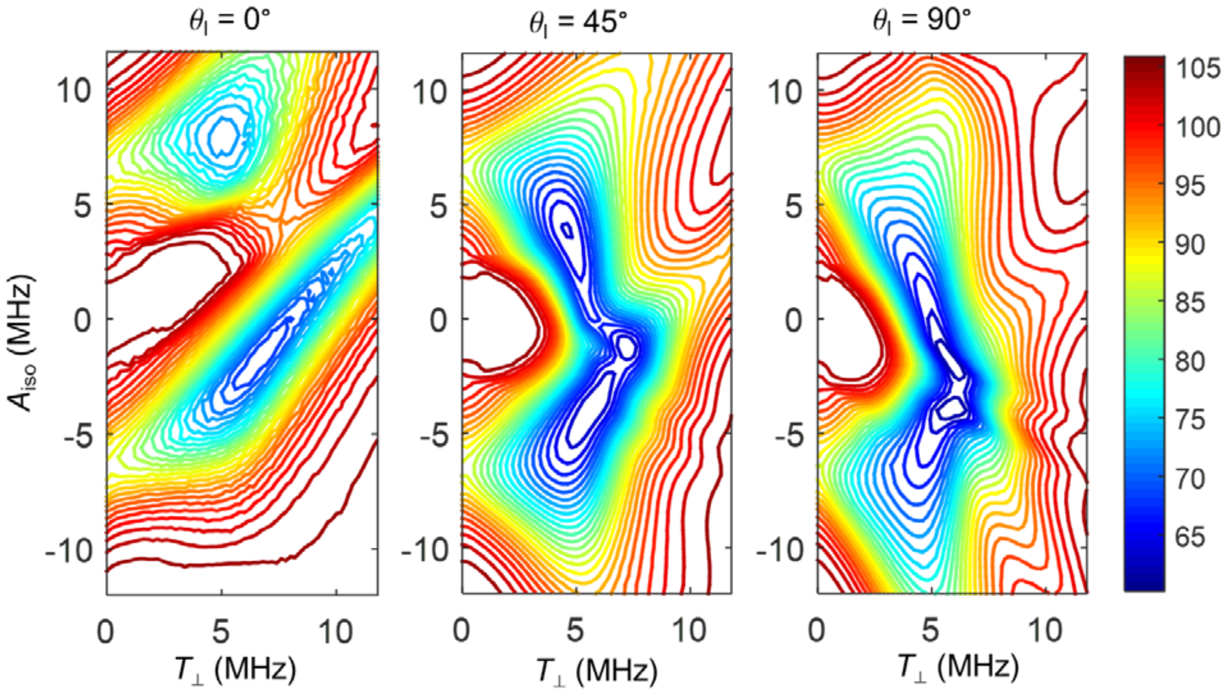

Supplementary Fig. 11. $\Delta\,(\theta_I,\,A_{iso},\,T_\perp)$ plotted as a function of θ_I, A_{iso}, and $T_\perp$ for ^{1}H HYSCORE region of hydrated Cu1-LT.

Surprisingly, a wide range of combinations of θ_I and A_{iso} produce multitude local minimums with $T_\perp$ ~ 5 – 6 MHz, likely due to the fact that the experimental spectrum comprises a distribution of different configurations at a certain Cu-H distance. Out of all local minimums, the simulated spectrum with $T_\perp$ = 5.6 MHz, A_{iso} = -4, and θ_I = 45° best matches the experimental spectrum, as shown in Supplementary Fig. 12.

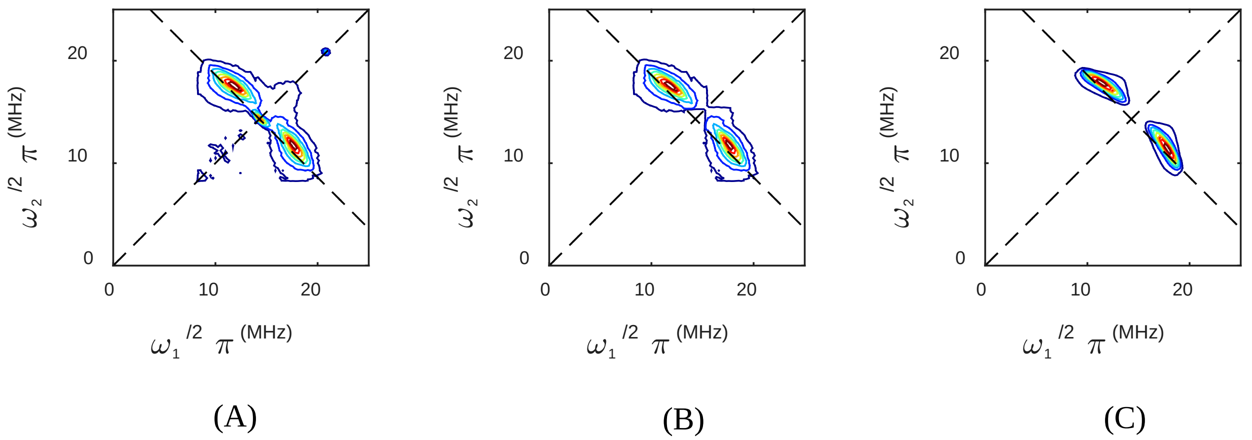

Supplementary Fig. 12 ^{1}H HYSCORE spectra for hydrated Cu1-LT. **A** Original experimental spectrum; **B** Experimental spectrum after removal of diagonal peaks resulting from coupling with distant protons and diagonal noises; **C** Simulated spectrum with ^{1}H $\omega_I /2\pi$ = 14.3059 MHz, τ = 128 ns, $T_\perp$ = 5.6 MHz, A_{iso} = -4 MHz, and θ_I = 45°. The line $\omega_2 = 2 \times \omega_I - \omega_1$ is shown as a visual guide for better comparison.

When simulating the ^{27}Al region, five parameters including the isotropic hyperfine parameter A_{iso}, the axial hyperfine parameter $T_\perp$, the orientation angle θ_I, the quadrupolar coupling constant QCC, and the asymmetry parameter η were varied with ranges and intervals shrinking step by step. In the final data matrix, A_{iso}, $T_\perp$, and QCC ranged in 0.2 – 2 MHz with an interval of 0.2 MHz, η = 0, 0.2, 0.4, 0.6, 0.8 and 1, and θ_I = 0°, 45° and 90°. Again the least-square residue was calculated and the spectrum with $T_\perp$ = 1.0 MHz, A_{iso} = 1.0 MHz, QCC = 1.4 MHz, η = 0, and θ_I = 90° was the best fit for the experimental ^{27}Al HYSCORE spectrum (Supplementary Fig. 13A).

The distance of Cu-H and Cu-Al can be calculated using the equation:

$$T_{\perp} = \frac{\mu_0}{4\pi} g_e g_n \beta_e \beta_n \frac{1}{r^3} \qquad (6)$$

Here, r is the distance between the electron spin and the nucleus. r(Cu-H) = 2.48 Å and r(Cu-Al) = 2.74 Å were obtained from $T_{\perp}$ (^{1}H) = 5.2 MHz and $T_{\perp}$ (^{27}Al) = 1.0 MHz.

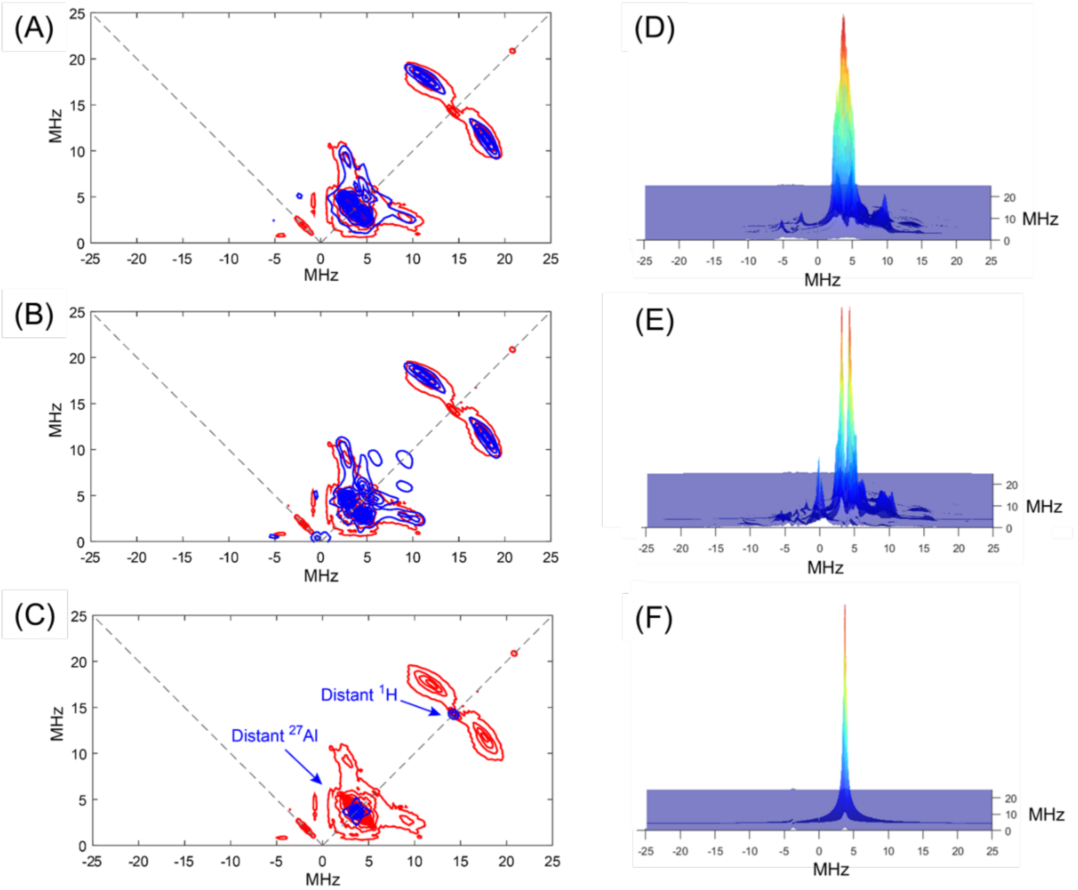

Supplementary Fig. 13 (A-C) Experimental HYSCORE spectrum (red) for Cu1-LT, and simulated spectrum (blue) with the simulation parameters for: (A) ^{1}H $\omega_I /2\pi$ = 14.3059 MHz, τ = 128 ns, $T_{\perp}$ = 5.6 MHz, A_{iso} = -4 MHz, and θ_I = 45°, and the parameters for ^{27}Al $\omega_I /2\pi$ = 3.7306 MHz, τ = 128 ns, $T_{\perp}$ = 1.0 MHz, A_{iso} = 1.0 MHz, QCC = 1.4 MHz, η = 0, and θ_I = 90°; (B) same parameters as in (A) but with two Al nuclei, θ_I = 52° for the second Al; (C) ^{1}H $T_{\perp}$ = 0.63 MHz, A_{iso} = 0, and θ_I = 45°, and ^{27}Al $T_{\perp}$ = 0.16 MHz, A_{iso} = 0 MHz, QCC = 1.4 MHz, η = 0, and θ_I = 90°, corresponding to a Cu-H or Cu-Al distance of 5 Å. (D-F) The surface plots of ^{27}Al HYSCORE simulated spectra using the parameters from (A-C), respectively.

However, two features of the experimental spectrum can only be poorly simulated using this approach. First, the off-diagonal peaks in the (-, +) quadrant have significantly different positions;

second, the central peak at around (ω_I, ω_I) has a different shape. Supplementary Fig. 13D shows that the center peak is much sharper than the experimental spectrum in Supplementary Fig. 9B. In order to compensate for the differences, a second Al with the same parameters but $\theta_I = 52°$ was introduced in the simulations. The combination of these two Cu-Al configurations allow for much improved simulations of the experimental peaks in the (-, +) quadrant (Supplementary Fig. 13B), and the peak splitting at (ω_I, ω_I) (Supplementary Fig. 13E). Supplementary Fig. 13C simulates the spectrum with distant ^{27}Al and ^{1}H (≥ 5 Å).

The nature of isolated Cu in Cu/SSZ-13 has been extensively studied in recent years. It is now generally agreed that two SCR active sites, i.e., $[Cu(OH)]^+$-Z and Cu^{2+}-2Z, where Z represents a framework negative charge, coexist in this catalyst.[6,7,12,17,18] HYSCORE simulations shown here demonstrate clearly that the same scenario holds for Cu/SAPO-34. Based on the simulation results, Cu1-F, Cu1-LT and Cu1-HT all exhibit combinations of Cu couplings to one Al and two Al; i.e., the presence of both $[Cu(OH)]^+$-Z and Cu^{2+}-2Z. On a semi-quantitative basis, Cu^{2+}-2Z/$[Cu(OH)]^+$-Z ratios in these samples follows the order: Cu1-HT > Cu1-LT ≈ Cu1-F. This is fully consistent with the fact that Cu^{2+}-2Z is thermodynamically more stable than $[Cu(OH)]^+$-Z; the latter converts to the former during high-temperature hydrothermal aging.[6,7,18,19] For Cu1-S, the sharp peak at ^{27}Al (ω_I, ω_I) in Supplementary Fig. 9D has a signal intensity an order of magnitude lower than the other three spectra, indicating that most Al ions are away from the Cu centers (≥ 5 Å). ^{1}H coupling patterns are similar for all spectra, but the intensity changes significantly, in the order of Cu1-F > Cu1-LT > Cu1-HT >> Cu1-S, suggesting the loss of $[Cu(OH)]^+$-Z active sites during low/high-temperature hydrothermal treating and during ambient temperature storage.

In addition, Supplementary Fig. 14A shows that a fair amount of $[Cu(OH)]^+$-Z sites in Cu1-F are still present after dehydration, but Cu-H coupling is below the detection limit in dehydrated

Cu1-S (Supplementary Fig. 14B), consistent with a complete CHA structural collapse and conversion of Cu into Cu-Al_2O_4-like species.

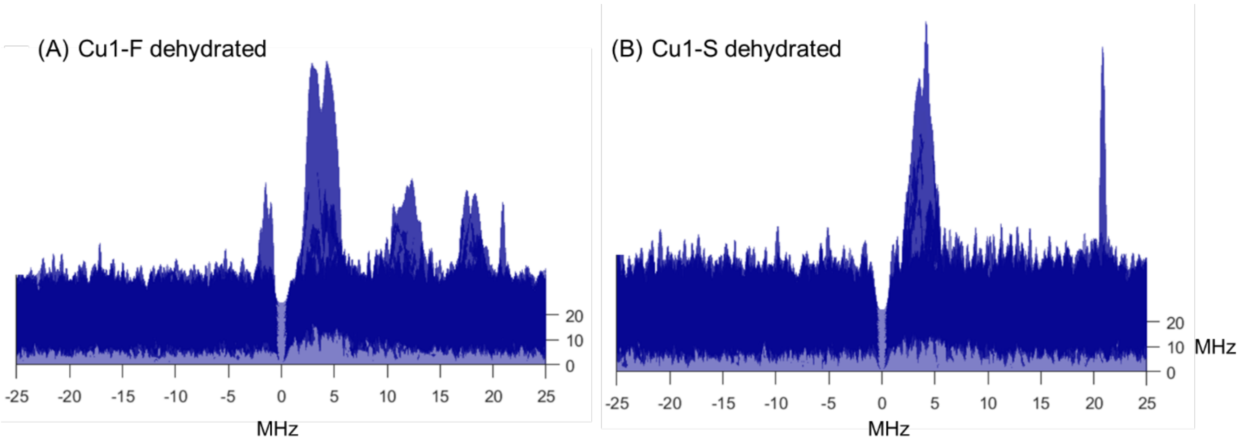

Supplementary Fig. 14 Experimental HYSCORE spectra of select dehydrated samples, plotted using the same color scale as in Supplementary Fig. 9. **A** Cu1-F; **B** Cu1-S. The signal to noise ratio is relatively low compared to the hydrated samples probably due to the slower motion of electron spins and nuclei due to the loss of water.

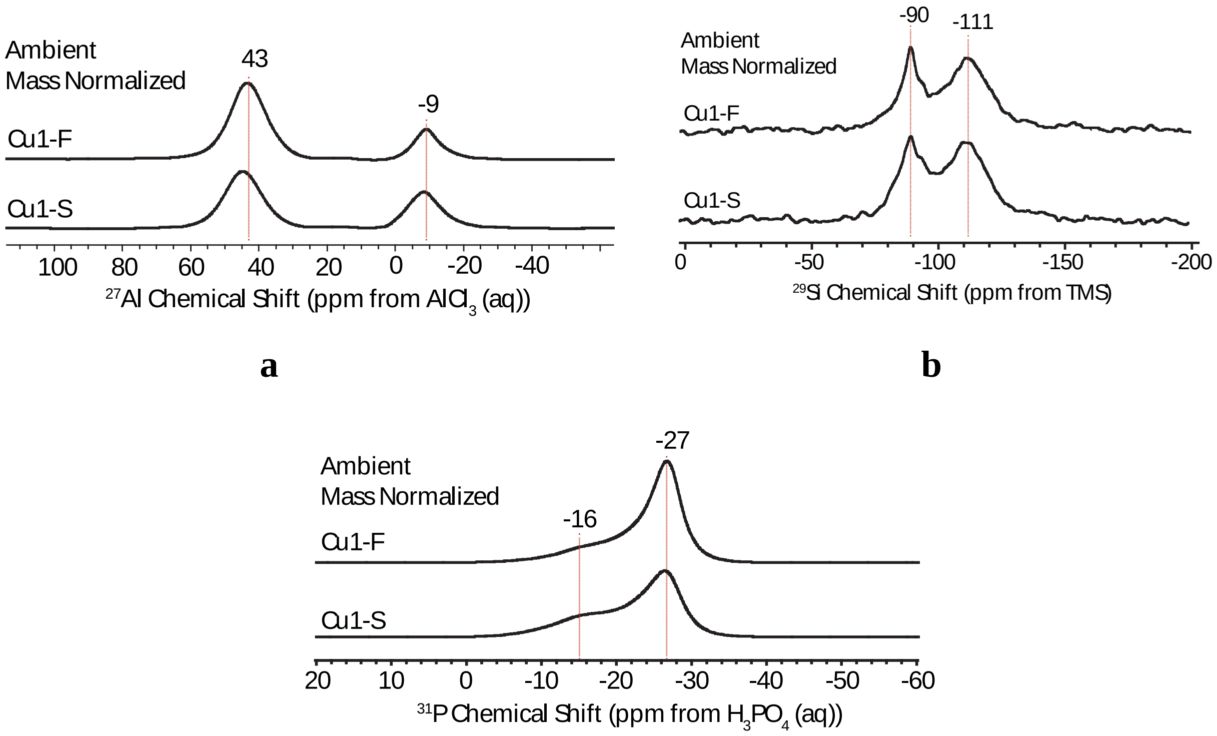

c

Supplementary Fig. 15 Solid-state NMR spectra for hydrated ambient Cu1-F and Cu1-S samples. **a** ^{27}Al; **b** ^{29}Si; **c** ^{31}P. All spectra are mass normalized for the purpose of direct comparison.

In Supplementary Fig. 15a, ^{27}Al signals centered at ~43 ppm are assigned to tetrahedrally coordinated framework aluminum atoms. Peaks at ~-9 ppm are attributed to octahedrally coordinated Al; e.g., framework Al coordinated with two H_2O molecules, or extraframework Al species.[3,20] A simple peak area comparison (signals mass normalized) demonstrates that the Cu1-F sample contains higher concentrations of tetrahedrally coordinated Al than Cu1-S; i.e., the Cu1-S sample is more defective. As shown in Supplementary Fig. 15b, both samples contain two prominent ^{29}Si features at -90 and -111 ppm, respectively. The former peak is assigned to framework Si with 4 Al in the first coordination sphere (i.e., $Si(OAl)_4$), and the latter to Si with 4

Si nearest neighbors as silica islands (i.e., $Si(OSi)_4$).[3,20] The presence of weaker $Si(OAl)_x(OSi)_{4-x}$ resonances between these two features cannot be ruled out. The $Si(OAl)_4$ resonance of the Cu1-S sample appears to be broader than that for Cu1-F; in particular, a shoulder feature appearing at ~86 ppm, attributable to tetrahedrally coordinated $Si(OAl)_4$ species with disordered local structures,[2,21] is evident. This again indicates that this sample is more defective. In Supplementary Fig. 15c, the -27 ppm ^{31}P resonance is attributed to framework P with 4 Al in the first coordination sphere (i.e., $P(OAl)_4$). The resonance at -16 ppm can be assigned to hydrated (i.e., $P(OAl)_4(H_2O)_x$), or defective (i.e., $P(OAl)_{4-x}(H_2O)_x$) P species.[20,22,23] The relative signal intensity comparison between the two samples is again consistent with a defective nature for Cu1-S.

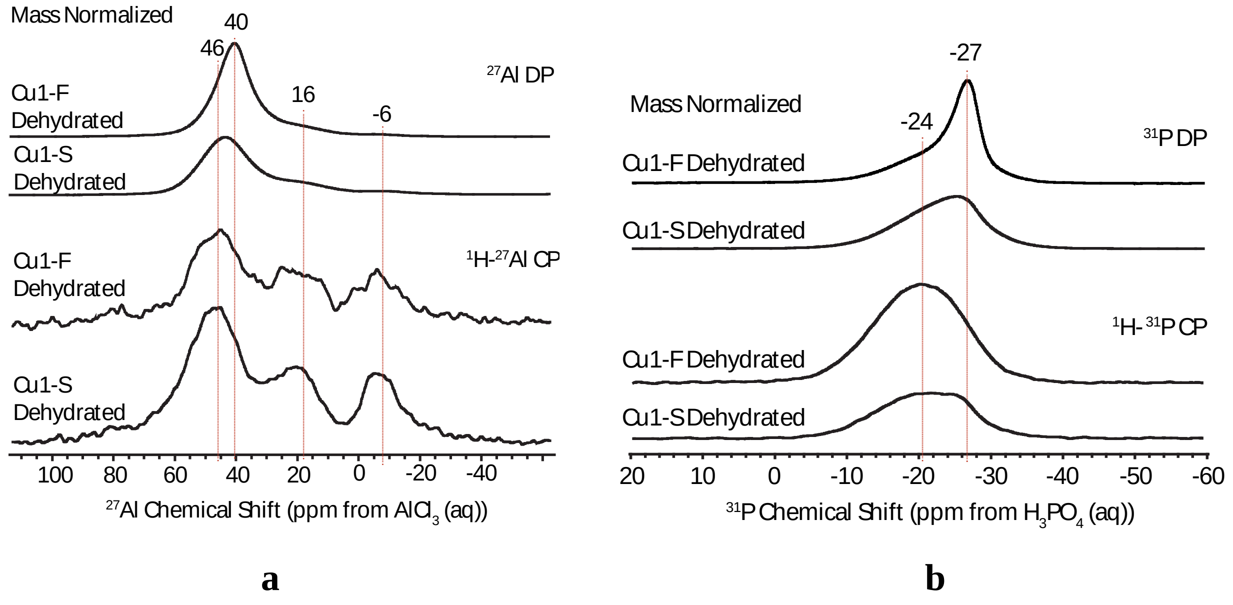

Supplementary Fig. 16 Solid-state NMR spectra for dehydrated Cu1-F and Cu1-S samples. **a** ^{27}Al and ^{1}H-^{27}Al; **b** ^{31}P and ^{1}H-^{31}P. All spectra are mass normalized for the purpose of direct comparison.

Supplementary Fig. 16a presents ^{27}Al DP and ^{1}H-^{27}Al cross polarization (CP) spectra of dehydrated Cu1-F and Cu1-S samples. For the Cu1-F sample, the DP spectrum displays tetrahedrally coordinated Al at 40 ppm, weak pentahedrally coordinated Al at 16 ppm, and extremely weak octahedrally coordinated Al at -6 ppm. In comparison to the spectrum of the hydrated sample, it appears that Si-O(H)-Al hydrolysis (which leads to formation of octahedrally coordinated Al) is largely reversible. For the Cu1-S sample, intensities for tetrahedrally coordinated Al decrease substantially as expected from the total CHA structure collapse as evidenced from in situ XRD (Supplementary Fig. 3b). The resonance also shifts to 46 ppm due to distortion. Surprisingly however, hardly any octahedrally coordinated Al is detected. NMR invisibility can have two causes: (1) strong interactions with paramagnetic Cu(II) sites; or (2) occupation of highly distorted sites.[24] Both causes are possible here. The ^{1}H-^{27}Al CP spectra of the samples provide important information on "defective" Al that couples more strongly with H in close vicinity (-OH or strongly bound H_2O), including distorted tetrahedral, pentahedral and octahedral Al sites at 46, 16 and -6 ppm, respectively. It seems clear that the Cu1-S sample has stronger H-Al coupling in comparison to Cu1-F, consistent with its more defective nature, and the important fact that H-Al coupling is preserved after total collapse of the CHA structure.

Supplementary Fig. 16b depicts the corresponding ^{31}P DP and ^{1}H-^{31}P CP spectra for these samples. Following the same analogy as ^{27}Al peak assignments, the resonance at -27 ppm is attributed to normal framework P and the feature at -24 to distorted P. The CP spectra demonstrate that distorted P couples more efficiently with H. Note that the Cu1-S sample shows lower ^{1}H-^{31}P coupling than Cu1-F, in contrast to ^{1}H-^{27}Al coupling. This suggests that H-containing moieties within the structure (-OH, strongly bound H_2O) preferentially interact with Al instead of P upon CHA structure collapse.

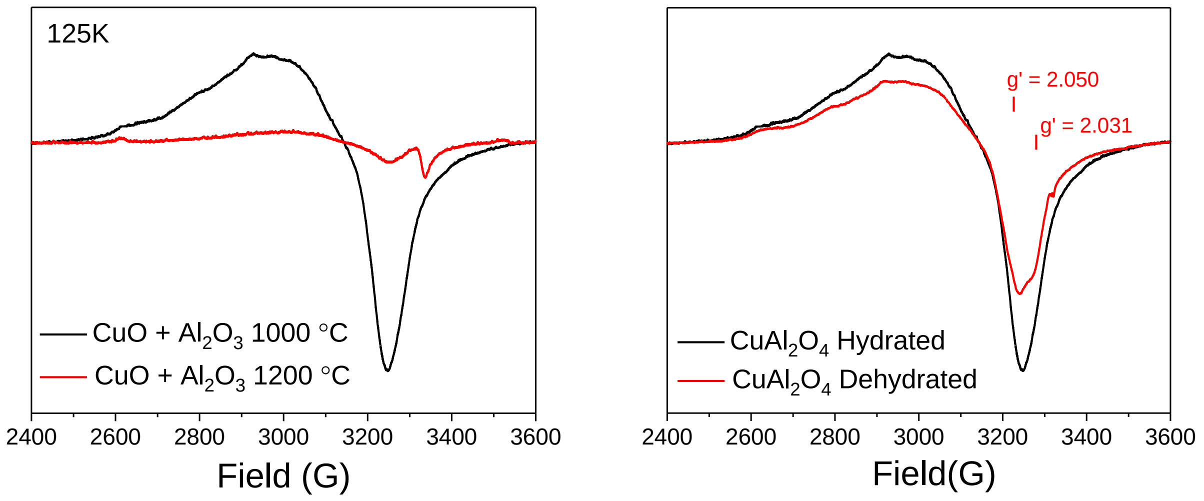

(a) (b)

Supplementary Fig. 17 Continuous wave (CW) EPR spectra of treated CuO + Al_2O_3 (~1% Cu) mixture. **a** spectra of the mixture calcined at 1000 and 1200 °C in static air for 12 h; **b** spectra of the mixture calcined at 1000 °C under both hydrated and dehydrated conditions. Measurements were conducted at 125 K.

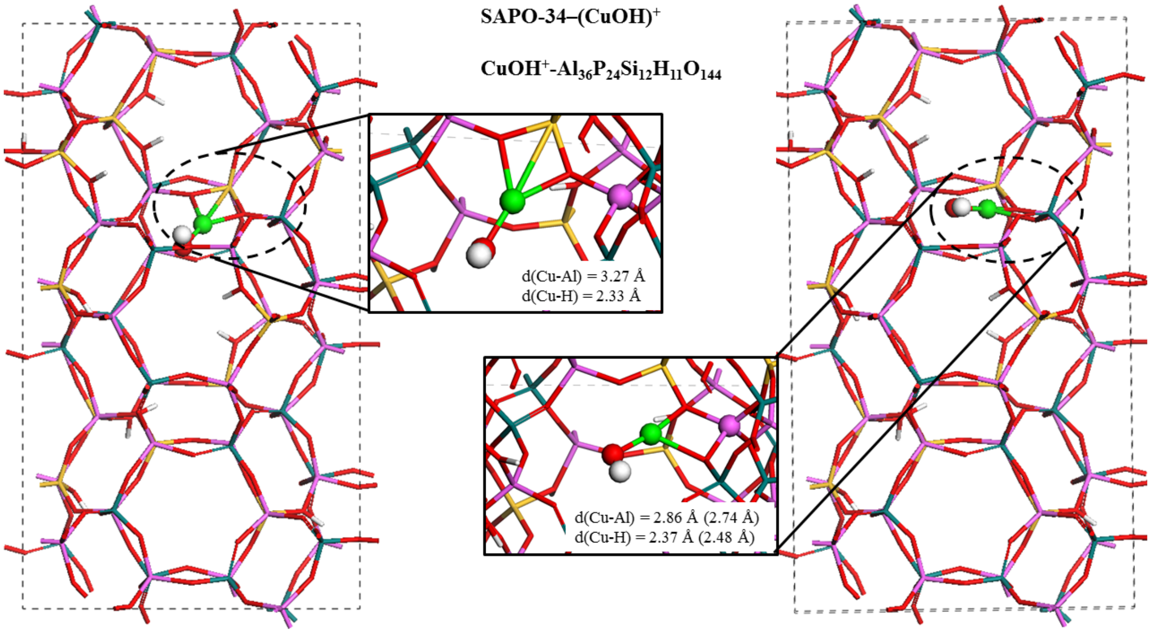

Supplementary Fig. 18 The optimized structures of the model Cu/SAPO-34 catalyst that mimics Cu1. The Cu, Si, P, Al, O and H atoms are distinguished using orange, yellow, green, magenta, red and white colors, respectively.

In the present work, two possible exchange positions for $[Cu(OH)]^+$-Z were tested. For the first positon, the $[Cu(OH)]^+$-Z binds to two lattice oxygen atoms directly connected with Si (left). In this configuration, the Cu-Al and Cu-H distances are 3.27 and 2.33 Å, respectively. In the second case, $[Cu(OH)]^+$-Z binds to two lattice oxygen atoms directly connected with Al (right). This configuration is thermodynamically more stable (10.4 kJ/mol) than the first one. Moreover, the simulated Cu-Al and Cu-H distances are 2.86 and 2.37 Å, respectively. These latter values are consistent with values derived from HYSCORE simulations (2.74 and 2.48 Å, respectively). As such, this second $[Cu(OH)]^+$-Z structure was adopted in the following DFT calculations.

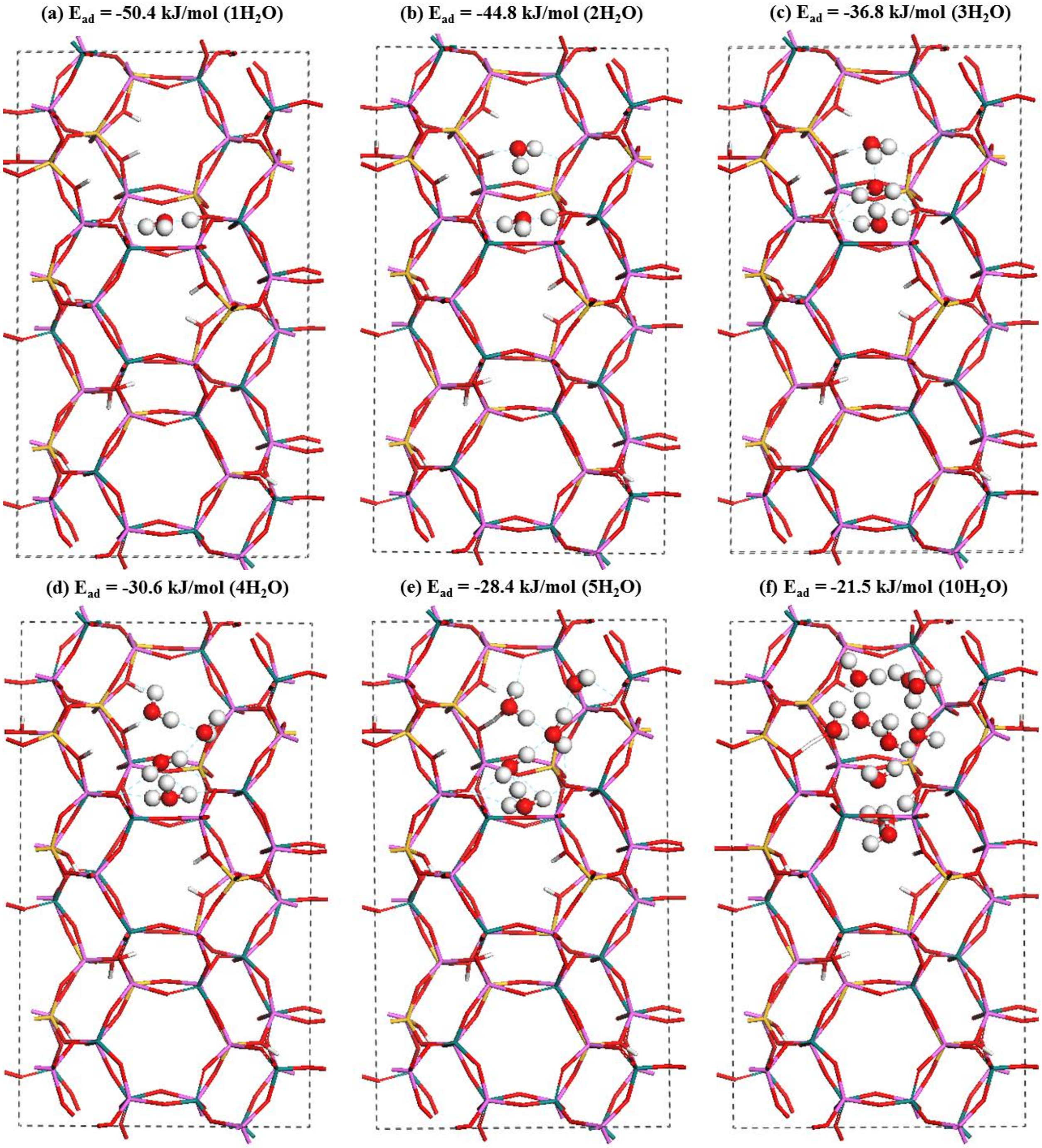

Supplementary Fig. 19 Water adsorption in the vicinity of an individual Brønsted acid site in SAPO-34, and the corresponding adsorption energies.

Up to ~10 H_2O molecules can be stabilized by each Brønsted acid site at 0 K. With further increases in number of the H_2O molecules, the newly added ones will migrate to neighboring Brønsted acid sites (not shown).

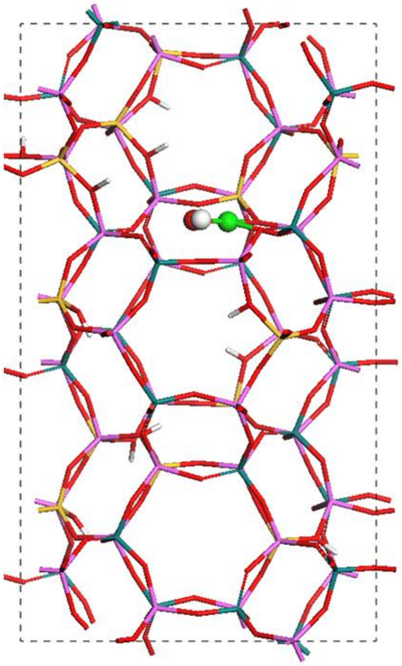

$[Cu(OH)]^+$

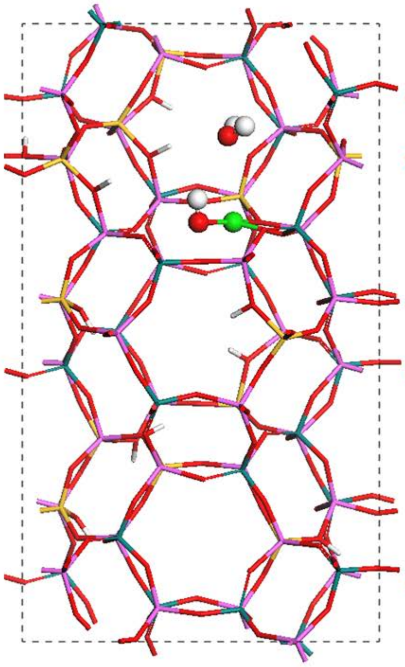

$[Cu(OH)]^+ + H_2O(g)$

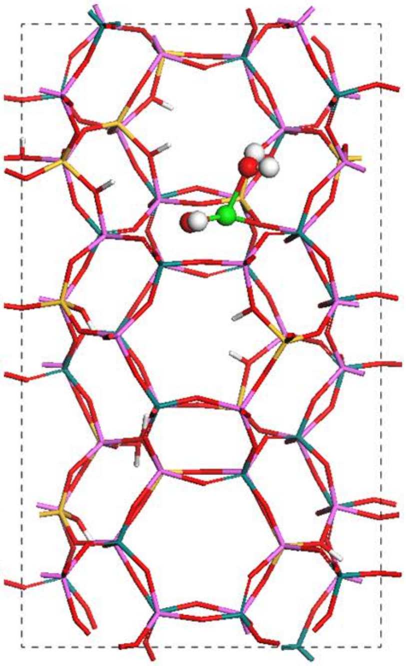

$[Cu(OH)]^+ \cdots H_2O^*$

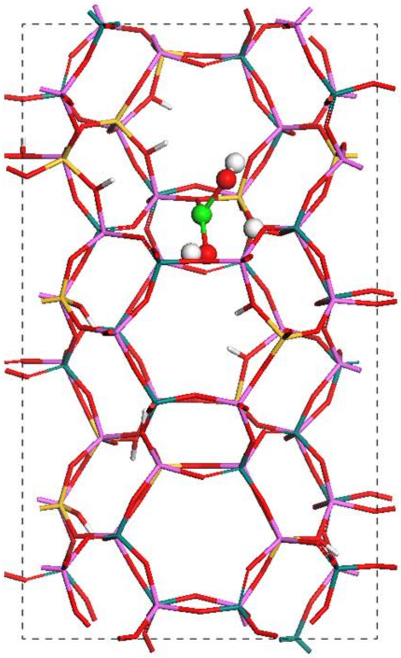

TS

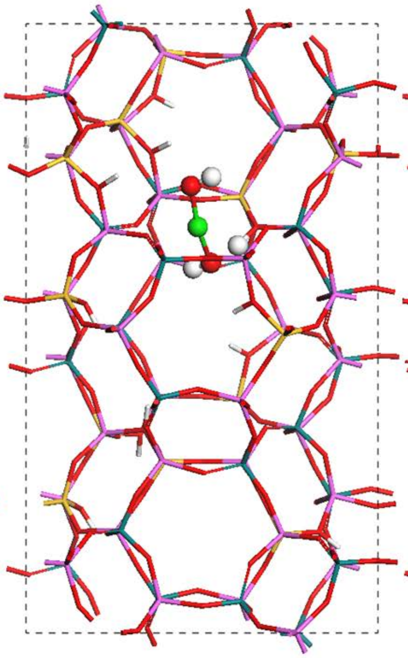

$Cu(OH)_2 + H^+$

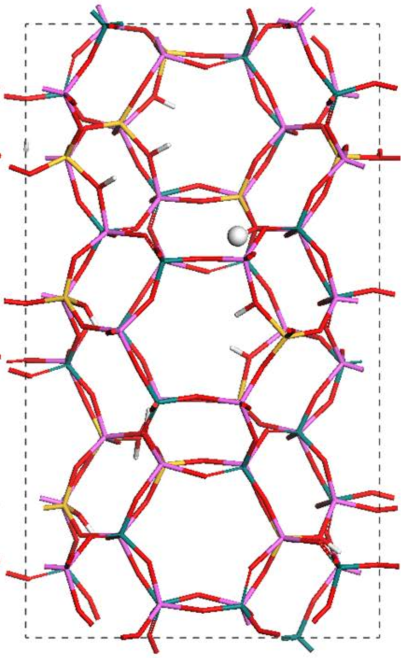

H^+

(continue)

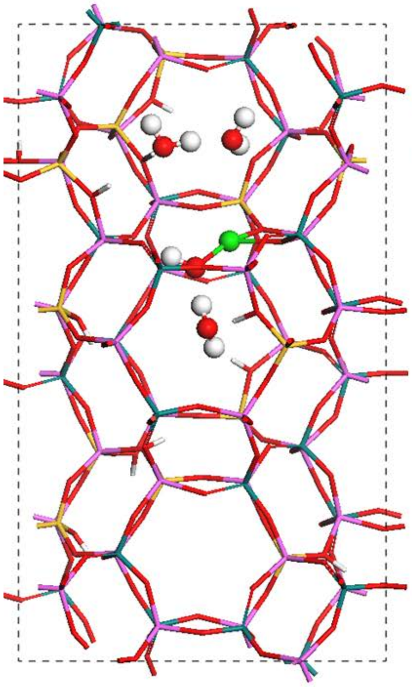

$[Cu(OH)^{+} + 3H_2O$

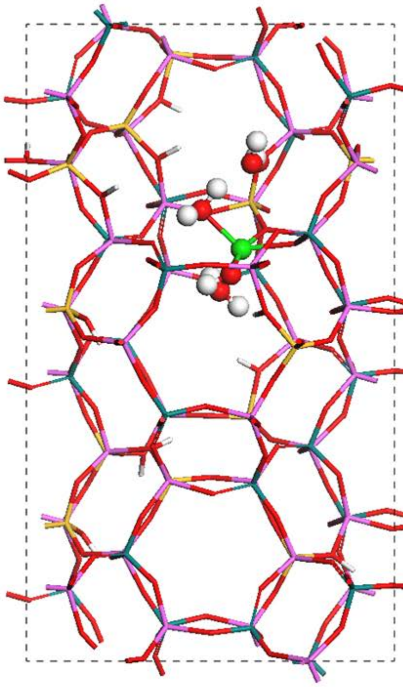

$[Cu(OH)^{+}\cdots H_2O^{*} + 2H_2O$

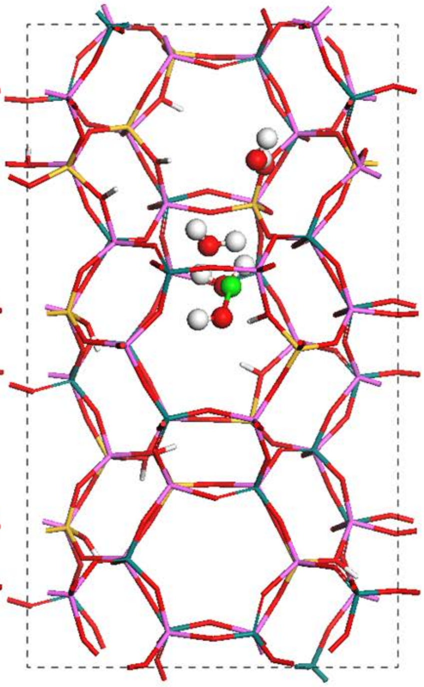

TS

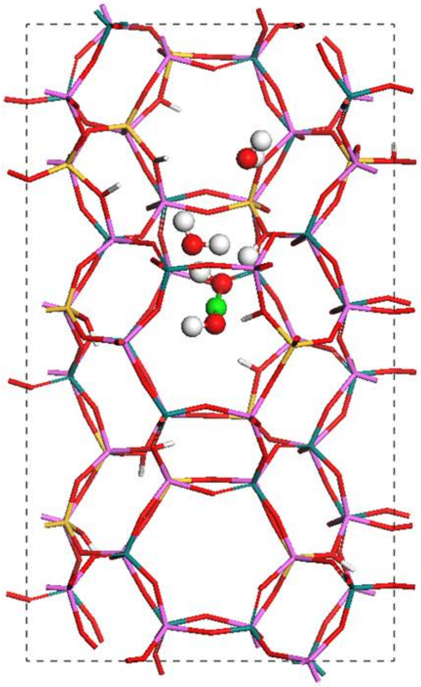

$Cu(OH)_2 + H^{+} + 2H_2O$

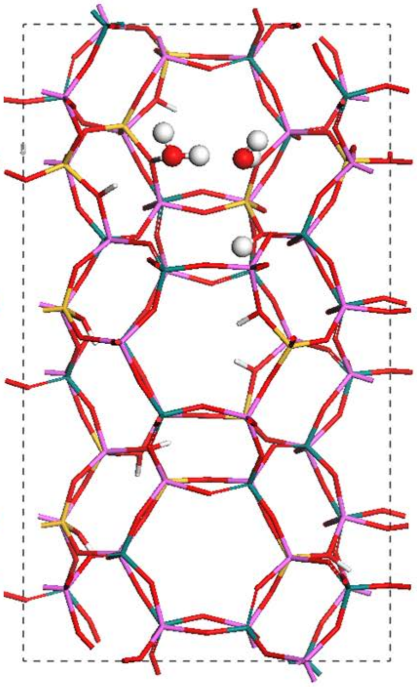

$H^{+} + 2H_2O$

(continue)

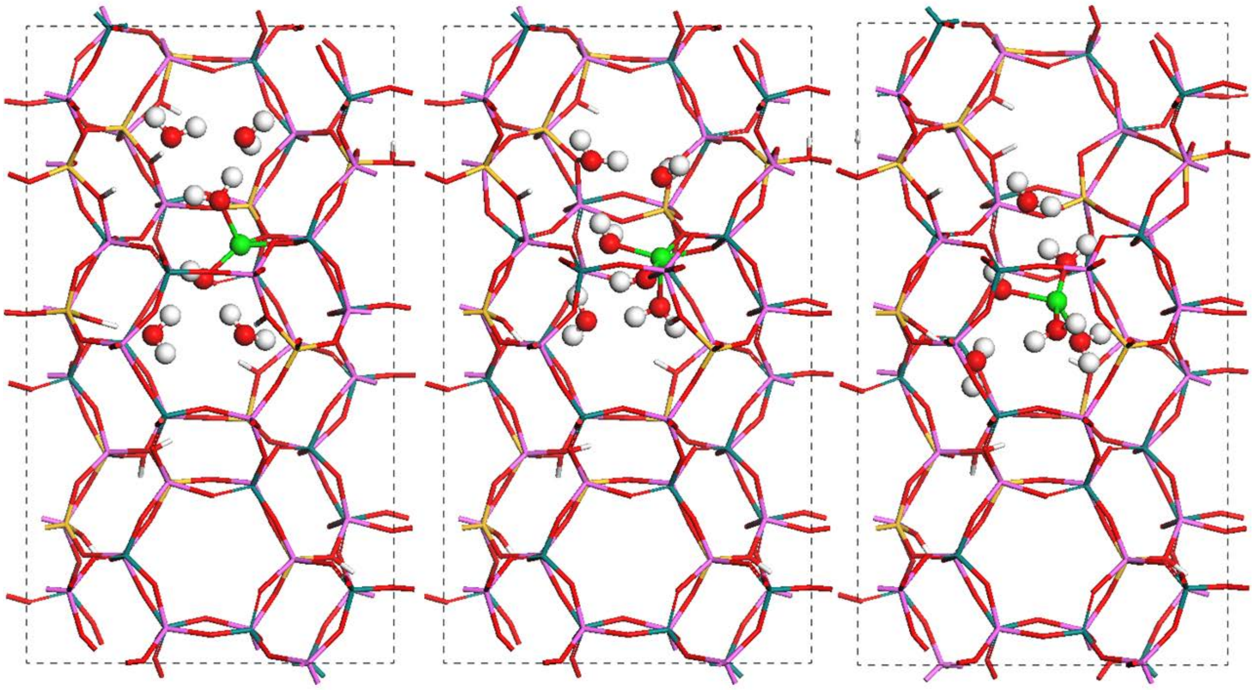

[Cu(OH)$^{+}$ + 5H_2O [Cu(OH)$^{+}\cdots$$H_2O$* + 4$H_2O$ TS

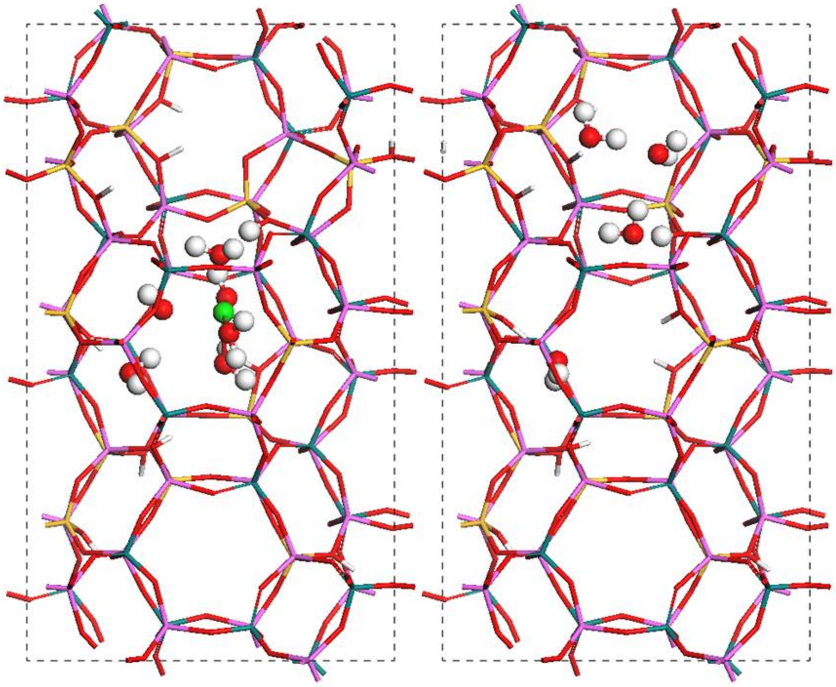

$Cu(OH)_2$ + H^{+} + 4H_2O H^{+} + 4H_2O

Supplementary Fig. 20 Initial, intermediate, transition state and final structures for the reaction processes shown in Fig. 5.

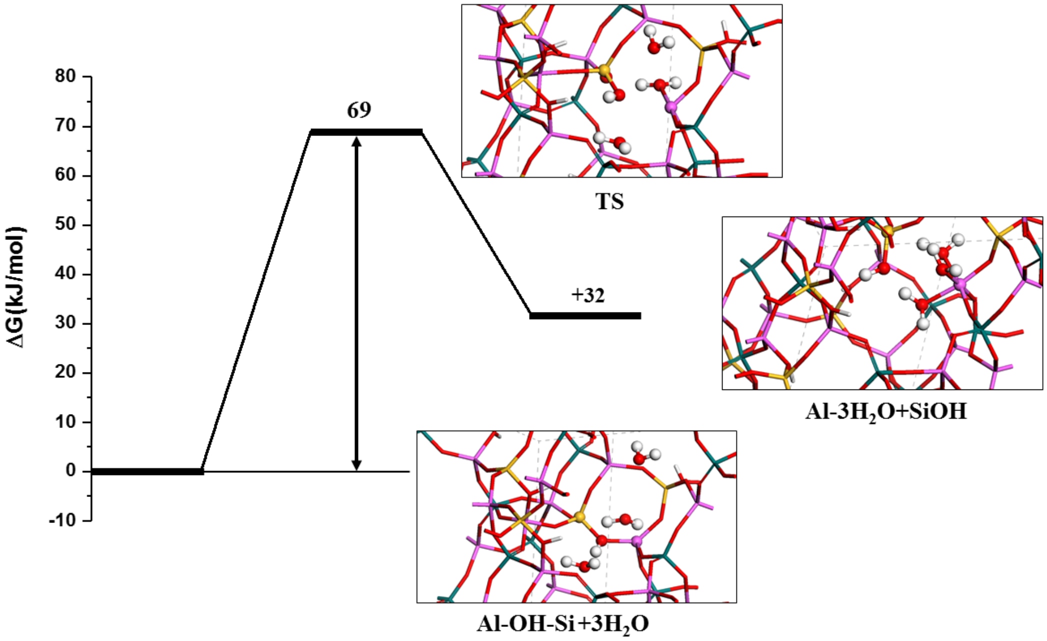

a

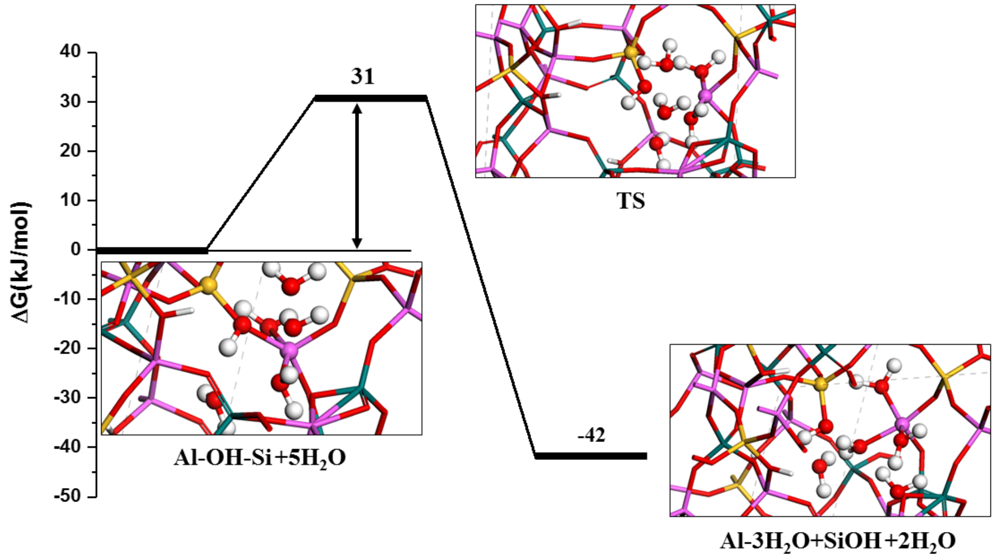

b

Supplementary Fig. 21 Hydrolysis of ≡Si-O(H)-Al≡ bonds in the presence of **a** 3 and **b** 5 water molecules.

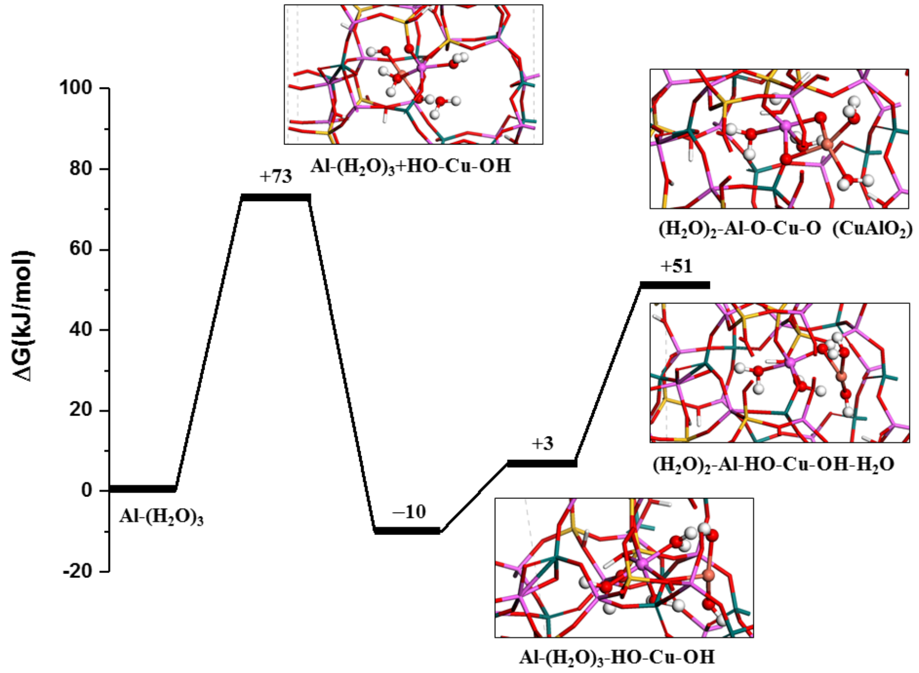

a

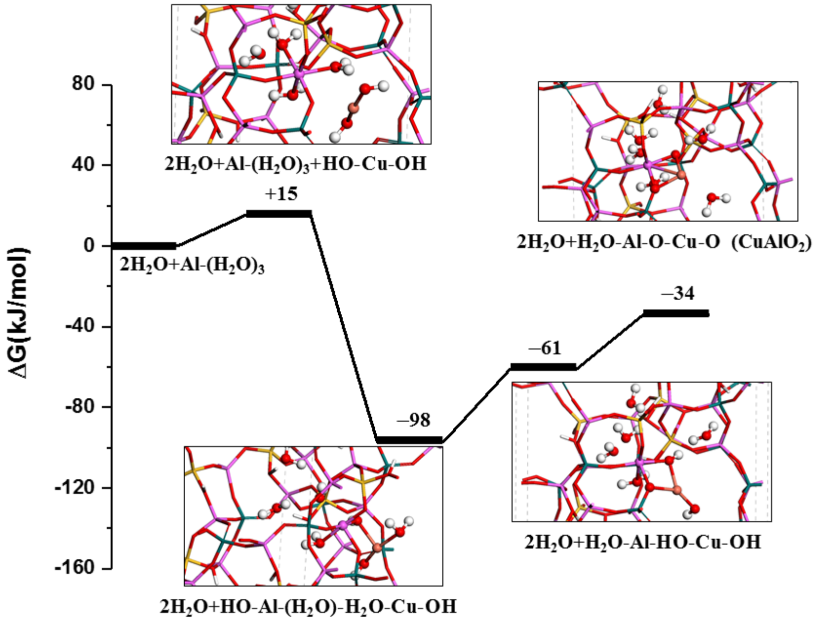

b

Supplementary Fig. 22 ≡Al(H_2O)$_3$ and Cu(OH)$_2$ interactions that form an =Al(μ-O_2)Cu intermediate as the precursor for $CuAl_2O_4$-like species **a** without and **b** with the assistance of 2 water molecules.

Supplementary References:

1 Vomscheid, R., Briend, M., Peltre, M. J., Man, P. P. & Barthomeuf, D. The Role of the Template in Directing the Si Distribution in SAPO Zeolites. *J Phys Chem* **98**, 9614-9618, (1994).

2 Briend, M., Vomscheid, R., Peltre, M. J., Man, P. P. & Barthomeuf, D. Influence of the Choice of the Template on the Short-Term and Long-Term Stability of SAPO-34 Zeolite. *J Phys Chem* **99**, 8270-8276, (1995).

3 Tan, J. *et al.* Crystallization and Si incorporation mechanisms of SAPO-34. *Micropor Mesopor Mat* **53**, 97-108, (2002).

4 Shen, W. L. *et al.* A study of the acidity of SAPO-34 by solid-state NMR spectroscopy. *Micropor Mesopor Mat* **158**, 19-25, (2012).

5 Gao, F. *et al.* Structure-activity relationships in NH_3-SCR over Cu-SSZ-13 as probed by reaction kinetics and EPR studies. *J Catal* **300**, 20-29, (2013).

6 Song, J. *et al.* Toward Rational Design of Cu/SSZ-13 Selective Catalytic Reduction Catalysts: Implications from Atomic-Level Understanding of Hydrothermal Stability. *ACS Catal* **7**, 8214-8227, (2017).

7 Gao, F. & Peden, C. H. F. Recent Progress in Atomic-Level Understanding of Cu/SSZ-13 Selective Catalytic Reduction Catalysts. *Catalysts* **8**, 140, (2018).

8 Wang, J. *et al.* Improvement of low-temperature hydrothermal stability of Cu/SAPO-34 catalysts by Cu^{2+} species. *J Catal* **322**, 84-90, (2015).

9 Wang, J. *et al.* The influence of silicon on the catalytic properties of Cu/SAPO-34 for NOx reduction by ammonia-SCR. *Appl Catal B-Environ* **127**, 137-147, (2012).

10 Wang, L., Li, W., Qi, G. S. & Weng, D. Location and nature of Cu species in Cu/SAPO-34 for selective catalytic reduction of NO with NH_3. *J Catal* **289**, 21-29, (2012).

11 Xue, J. J. *et al.* Characterization of copper species over Cu/SAPO-34 in selective catalytic reduction of NOx with ammonia: Relationships between active Cu sites and de-NOx performance at low temperature. *J Catal* **297**, 56-64, (2013).

12 Gao, F. *et al.* Effects of Si/Al ratio on Cu/SSZ-13 NH_3-SCR catalysts: Implications for the active Cu species and the roles of Bronsted acidity. *J Catal* **331**, 25-38, (2015).

13 Vennestrom, P. N. R. *et al.* Influence of lattice stability on hydrothermal deactivation of Cu-ZSM-5 and Cu-IM-5 zeolites for selective catalytic reduction of NOx by NH_3. *J Catal* **309**, 477-490, (2014).

14 Giordanino, F. *et al.* Characterization of Cu-exchanged SSZ-13: a comparative FTIR, UV-Vis, and EPR study with Cu-ZSM-5 and Cu-beta with similar Si/Al and Cu/Al ratios. *Dalton T* **42**, 12741-12761, (2013).

15 Snyder, B. E. R., Bols, M. L., Schoonheydt, R. A., Sels, B. F. & Solomon, E. I. Iron and Copper Active Sites in Zeolites and Their Correlation to Metalloenzymes. *Chem Rev* **118**, 2718-2768, (2018).

16 Stoll, S. & Schweiger, A. EasySpin, a comprehensive software package for spectral simulation and analysis in EPR. *Journal of Magnetic Resonance* **178**, 42-55, (2006).

17 Beale, A. M., Gao, F., Lezcano-Gonzalez, I., Peden, C. H. F. & Szanyi, J. Recent advances in automotive catalysis for NOx emission control by small-pore microporous materials. *Chemical Society Reviews* **44**, 7371-7405, (2015).

18 Gao, F. & Szanyi, J. On the hydrothermal stability of Cu/SSZ-13 SCR catalysts. *Appl Catal A-Gen* **560**, 185-194, (2018).

19 Luo, J. Y. *et al.* New insights into Cu/SSZ-13 SCR catalyst acidity. Part I: Nature of acidic sites probed by NH_3 titration. *J Catal* **348**, 291-299, (2017).
20 Buchholz, A., Wang, W., Arnold, A., Xu, M. & Hunger, M. Successive steps of hydration and dehydration of silicoaluminophosphates H-SAPO-34 and H-SAPO-37 investigated by in situ CF MAS NMR spectroscopy. *Micropor Mesopor Mat* **57**, 157-168 (2003).
21 Martens, J. A., Grobet, P. J. & Jacobs, P. A. Catalytic Activity and Si, Al, P Ordering in Microporous Silicoaluminophosphates of the SAPO-5, SAPO-11, and SAPO-37 Type. *J Catal* **126**, 299-305, (1990).
22 Watanabe, Y., Koiwai, A., Takeuchi, H., Hyodo, S. A. & Noda, S. Multinuclear NMR Studies on the Thermal-Stability of SAPO-34. *J Catal* **143**, 430-436, (1993).
23 Vomscheid, R. *et al.* Reversible Modification of the Si Environment in Template-Free SAPO-34 Structure Upon Hydration Dehydration Cycles Below ca. 400 K. *J Chem Soc Chem Comm*, 544-546, (1993).
24 Prodinger, S. *et al.* Sub-micron Cu/SSZ-13: Synthesis and application as selective catalytic reduction (SCR) catalysts. *Appl Catal B-Environ* **201**, 461-469, (2017).
