# Supplementary material for: Unraveling the mysterious failure of Cu/SAPO-34 selective catalytic reduction catalysts
Source: Nat Commun. 2019 Mar 8;10:1137. doi: 10.1038/s41467-019-09021-3 (PMC6408507; doi:10.1038/s41467-019-09021-3)
Supplement: Supplementary file 2 — Peer Review [file 41467_2019_9021_MOESM2_ESM.pdf]

Reviewers' comments:

Reviewer #1 (Remarks to the Author):

This manuscript addresses the well-known failure of Cu/SAPO-34 catalyst for the SCR upon the low-temperature exposure to H2O. The topic itself has important meaning not only academically, but also practically. Authors performed lots of experiments including activity measurement, various characterizations, and DFT calculation. All combined results clearly indicated the deactivation mechanism of Cu species within the SAPO-34 pore. Moreover, they applied novel 2-D EPR techniques to elucidate the behavior of copper species during the deactivation. Since the overall quality of the manuscript meets the standards of Nat. Comm., reviewer recommends the publication after addressing a few things in the following.

1) The authors studied just two samples which are low Cu loading and high Cu loading. Although two samples represent failed and not-failed ones, respectively, it might be highly informative to provide information about the transition Cu loading. Hence, it is strongly recommended to perform a (few) sample(s) in the middle Cu loading.

2) Authors simulated the amount of H2O in the zeolite pore as shown in Fig. 4 and Fig. S19. How about performing H2O TPD to estimate the real amount of H2O in the samples?

3) Since authors claimed that 2-D EPR technique is a powerful tool to elucidate the behavior of Cu species in Cu-SAPO-34, it is recommended to add the explanation to the manuscript regarding the comparison between Cu-SSZ_13 and Cu-SAPO-34 during the deactivation. It might be very interesting to
the people in the area.

Reviewer #2 (Remarks to the Author):

Understanding the deactivation mechanism of zeolite catalyst for NH3-SCR is one of the important research topics in zeolite science field. Especially deactivation of SAPO34 at low temperatures with the existence of water is mysterious and many automotive companies are facing this problem. In this research authors prepared model Cu/SAPO34 catalyst and proposed the deactivation steps by combination of kinetic, characterization and simulation studies.
This contribution attracts great interest from chemists in a wide range of fields such as materials chemistry and catalytic science. The interpretation of the data as well as the discussion is reasonable and convincing. Also the authors have made clear explanations what changes proceeded during catalyst deactivation on Cu/SAPO-34 on an atomic scale – the one that can't be achieved without so many experiments and characterizations-- that justifies pub in Nature Communications. Therefore, I suggest that this manuscript be accepted in the present form. Also please see below.

Comments:
Please provide SEM and Photo images of samples before and after SCR test for better understanding.

Reviewer #3 (Remarks to the Author):

The manuscript by Wang and co-authors investigated the long-known, but mechanistically unresolved low-temperature hydrothermal destruction of Cu-SAPO-34 NH3-SCR catalysts used commercially for automotive DeNOx. By integrating reaction kinetics, comprehensive structural characterization (NH3-TPD, H2-TPR, XRD, 1D/2D EPR, NMR, etc.) and systematic DFT calculations, the authors unveiled that condensed water molecules facilitated the formation of =Al(μ-O2)Cu, a precursor to CuAl2O4 which

eventually leads to the unusual failure of Cu/SAPO-34 . The work is highly important for both real application in automotive emission control and fundamental understanding of the dynamic transformation of zeolite-confined single-site catalysts, and therefore is of great interest for specialists working in related fields. In general, the experiments and theoretical calculations were well designed, leading to reliable and important insights into the destruction mechanisms of Cu-SAPO-34. The application of 2D EPR to explore the electronic interactions between Cu sites and nearby H/Al atoms is impressive. The manuscript was very well written, and, in the reviewer's opinion, can be published in its current form in Nature Communications.

There are only some comments from the reviewer as below:
From the DFT-computed structures shown in Fig. S20, the Cu site seems to undergo dynamic local motion upon interaction with H2O. Do the authors think if the Cu mobility plays a role in the destruction of SAPO-34 structure? Upon the introduction of a second Cu atom into the super cell (maybe the case for the Cu2 series catalysts in this study), will there be any cation-cation repulsive interaction decreasing the mobility of H2O-solvated Cu species, thus suppressing somehow their attack to the SAPO-34 framework and eventually the structural destruction? Any discussions/comments on these issues by the authors are desired, although not really necessary for the publication of this manuscript.

Some technical issues:
1) The authors should comment on the humidity of "typical ambient lab environment" (Page 4) for sample storage.
2) Method for the quantification of EPR active Cu species using solution Cu-imide complexes is not described in ref. 19 (Page 6). The authors should check if they cited a wrong reference.
3) Fig. S7/S8: the reviewer suggests to label the different features with dashed lines crossing the peaks for self-explaining of the figures.
4) NH3-TPD/H2-TPR should be defined upon first appearance in the main article.

Reviewers' comments:

Reviewer #1 (Remarks to the Author):

This manuscript addresses the well-known failure of Cu/SAPO-34 catalyst for the SCR upon the low-temperature exposure to H_2O. The topic itself has important meaning not only academically, but also practically. Authors performed lots of experiments including activity measurement, various characterizations, and DFT calculation. All combined results clearly indicated the deactivation mechanism of Cu species within the SAPO-34 pore. Moreover, they applied novel 2-D EPR techniques to elucidate the behavior of copper species during the deactivation. Since the overall quality of the manuscript meets the standards of Nat. Comm., reviewer recommends the publication after addressing a few things in the following.

1) The authors studied just two samples which are low Cu loading and high Cu loading. Although two samples represent failed and not-failed ones, respectively, it might be highly informative to provide information about the transition Cu loading. Hence, it is strongly recommended to perform a (few) sample(s) in the middle Cu loading.

We thank the reviewer for this suggestion, which is certainly very useful for us to learn more about the transition from less stable to more stable catalysts. However we decide not to add this new component to the current paper for the following reasons:

(1) Similar work has already been conducted by researchers from Tianjin University (*Wang, et al. "Improvement of low-temperature hydrothermal stability of Cu/SAPO-34 catalysts by Cu^{2+} species" Journal of Catalysis 322 (2015):84-90),* where the authors systematically investigated the effect of Cu loading (from 0 to 6.78 wt%) on low-temperature hydrothermal stability of Cu/SAPO-34 catalysts.

(2) The main focus of the current study is to provide an atomic-level understanding of the water induced low-temperature deactivation mechanism. It is our belief that this goal is only achievable with the aid of well-defined model Cu/SAPO-34 catalysts with fully isolated Cu-ions. Samples with intermediate Cu loadings will have Cu dispersions less ideal for such a research goal to be realized.

In any case, we will still take this comment seriously, and prepare catalysts with intermediate Cu loadings, and research on their low-temperature water sensitivity, and report the results later.

2) Authors simulated the amount of H_2O in the zeolite pore as shown in Fig. 4 and Fig. S19. How about performing H_2O TPD to estimate the real amount of H_2O in the samples?

We thank the reviewer for this great suggestion. The MKS FTIR detector we use for gas analysis is not accurate enough for detecting H_2O desorption at ppm levels. Instead of H_2O-TPD, we used weight measurements to quantify weight loss of fully hydrated ambient samples in dehydration, where dehydration is conducted at 250 °C overnight under high vacuum. The results are tabulated in the following Table.

Sample	Cu1-F	Cu1-LT	Cu1-HT	Cu1-S	Cu2-F	Cu2-LT	Cu2-HT
Weight loss during dehydration (mg/g)	178	81	103	180	223	188	207

Note that these weight losses are consistent with micropore volumes of the samples displayed in Table 1, where samples with higher micropore volumes display higher weight losses during dehydration.

On page 13 of the manuscript, we described that each hexagonal unit cell can store up to ~15 molecules from DFT calculations. From the unit cell stoichiometry shown in Table S1, one can readily estimate molecular weight of each such unit cell as ~2205 g/mol for Cu1-F. Then the weight loss due to water desorption with 15 adsorbed H_2O per unit cell is ~110 mg/g. The fact that the measured weight loss is even larger than that suggests that not only micropores are saturated with water, other larger voids and framework defects are all capable of storing water at ambient

conditions. From the comparisons shown above, our DFT calculations regarding H_2O stabilization by Brønsted acid sites below ~100 °C are readily justified.

On pages 13-14 of the revised manuscript, the following sentences have been added:

(1) water adsorption by Cu/SAPO-34 must be associated with Brønsted acid sites, Cu-ions and other minor defect sites. At low Cu loadings (e.g., Cu1), Brønsted acid sites are considered as the main contributor for H_2O adsorption.
(2) Such estimations can be readily verified experimentally. Desorption of 15 H_2O molecules from one unit cell corresponds to ~10% weight loss; this value is consistent with typical weight losses of ~10-20% in Cu/SAPO-34 dehydration.

3) Since authors claimed that 2-D EPR technique is a powerful tool to elucidate the behavior of Cu species in Cu-SAPO-34, it is recommended to add the explanation to the manuscript regarding the comparison between Cu-SSZ_13 and Cu-SAPO-34 during the deactivation. It might be very interesting to the people in the area.

We fully agree with the reviewer that comparison between Cu/SSZ-13 and Cu/SAPO-34 is an interesting topic in SCR research. As a matter of fact, we are currently working on 2D EPR and DFT investigations of low-temperature water treated Cu/SSZ-13 and Cu/SAPO-34 catalysts in order to reveal why only Cu/SAPO-34 deactivates under such conditions. Since the work is still ongoing, we plan to report the results separately later.

Reviewer #2 (Remarks to the Author):

Understanding the deactivation mechanism of zeolite catalyst for NH_3-SCR is one of the important research topics in zeolite science field. Especially deactivation of SAPO-34 at low temperatures with the existence of water is mysterious and many automotive companies are facing this problem. In this research authors prepared model Cu/SAPO-34 catalyst and proposed the deactivation steps by combination of kinetic, characterization and simulation studies.
This contribution attracts great interest from chemists in a wide range of fields such as materials chemistry and catalytic science. The interpretation of the data as well as the discussion is reasonable and convincing. Also the authors have made clear explanations what changes proceeded during catalyst deactivation on Cu/SAPO-34 on an atomic scale – the one that can't be achieved without so many experiments and characterizations-- that justifies pub in Nature Communications. Therefore, I suggest that this manuscript be accepted in the present form. Also please see below.

Comments:
Please provide SEM and Photo images of samples before and after SCR test for better understanding.

We thank the review for this suggestion. However, we would like to point out that as bulk techniques, SEM or helium ion imaging cannot provide atomic-level knowledge regarding catalyst deactivation. For example, in a previous study by *Yu, et al. "NH_3-SCR over Cu/SAPO-34 catalysts with various acid contents and low Cu loading" Catalysis Science & Technology 3. 12 (2013): 3234-3241*, the authors failed to observe any morphological differences between samples before and after solution ion-exchange, even though it is well-known that some level of degradation of the SAPO-34 support certainly occurs during solution ion-exchange. We do although, use another bulk technique XRD to examine samples before and after SCR tests routinely. Except for the Cu1-S sample which collapses during sample heating, other samples do not show obvious differences in their XRD patterns before and after SCR tests.

To address the point that the catalysts do not degrade continuously during SCR tests, the following sentence has been added on page 6 that reads "It is important to note that the rate losses described above are caused by the water treatments prior to SCR tests. During the short SCR tests,

no catalyst deactivation has been observed."

Reviewer #3 (Remarks to the Author):

The manuscript by Wang and co-authors investigated the long-known, but mechanistically unresolved low-temperature hydrothermal destruction of Cu-SAPO-34 NH_3-SCR catalysts used commercially for automotive DeNOx. By integrating reaction kinetics, comprehensive structural characterization (NH_3-TPD, H_2-TPR, XRD, 1D/2D EPR, NMR, etc.) and systematic DFT calculations, the authors unveiled that condensed water molecules facilitated the formation of =Al(μ-O2)Cu, a precursor to $CuAl_2O_4$ which eventually leads to the unusual failure of Cu/SAPO-34 . The work is highly important for both real application in automotive emission control and fundamental understanding of the dynamic transformation of zeolite-confined single-site catalysts, and therefore is of great interest for specialists working in related fields. In general, the experiments and theoretical calculations were well designed, leading to reliable and important insights into the destruction mechanisms of Cu-SAPO-34. The application of 2D EPR to explore the electronic interactions between Cu sites and nearby H/Al atoms is impressive. The manuscript was very well written, and, in the reviewer's opinion, can be published in its current form in Nature Communications.

There are only some comments from the reviewer as below:
From the DFT-computed structures shown in Fig. S20, the Cu site seems to undergo dynamic local motion upon interaction with H_2O. Do the authors think if the Cu mobility plays a role in the destruction of SAPO-34 structure? Upon the introduction of a second Cu atom into the super cell (maybe the case for the Cu2 series catalysts in this study), will there be any cation-cation repulsive interaction decreasing the mobility of H_2O-solvated Cu species, thus suppressing somehow their attack to the SAPO-34 framework and eventually the structural destruction? Any discussions/comments on these issues by the authors are desired, although not really necessary for the publication of this manuscript.

We are grateful to the reviewer for providing such useful comments that spur further discussions. First of all, the protective role for isolated Cu(II) ions is obvious, demonstrated both by the work from Tianjin (*Wang, et al. "Improvement of low-temperature hydrothermal stability of Cu/SAPO-34 catalysts by Cu^{2+} species" Journal of Catalysis 322 (2015):84-90*), and the present study. In the work by Wang et al., the authors discovered that Cu/SAPO-34 catalysts free from low-temperature water attack contain saturated amounts of ~2.2 wt% isolated Cu(II) ions as determined via EPR. Such loadings correspond to ~1 isolated Cu(II) ion per hexagonal unit cell. This suggests two things: (1) repulsive interactions between mobile water-solvated Cu(II) ions preclude the coexistence of more than one such ions in each unit cell; (2) when each unit cell contains one isolated Cu(II) ion, it is well protected from low-temperature water attack. For our Cu1-F sample, isolated Cu(II) loading is 0.7 wt%, corresponding to one Cu(II) in every 4 unit cells, i.e., 3 of 4 unit cells are never protected in low-temperature water attack. It can be anticipated that unit cells without Cu(II) protection will yield first from water attack, leaving unit cells contain Cu(II) much more vulnerable to water attack.

On page 16 of the revised manuscript, the following descriptions has been added that read " In the work by Wang et al.,[8] the authors discovered that Cu/SAPO-34 catalysts free from low-temperature water attack contain saturated amounts of ~2.2 wt% isolated Cu(II) ions as determined via EPR. Such loadings correspond to ~1 isolated Cu(II) ion per hexagonal unit cell. This suggests that: (1) repulsive interactions between mobile water-solvated Cu(II) ions preclude the coexistence of more than one such ions in each unit cell; (2) when each unit cell contains one such isolated Cu(II) ion, it is well protected from low-temperature water attack. Our Cu1-F sample contains an isolated Cu(II) loading of 0.7 wt%, corresponding to one Cu(II) ion in every 4 unit cells, i.e., 3 of 4 unit cells are unprotected from low-temperature water attack. It can be anticipated that unit cells without Cu(II) protection will deform first (not necessarily collapse since the pores are filled with water) from low-temperature water attack, leaving unit cells containing Cu(II) much more vulnerable to water attack. Therefore,

the “one isolated Cu(II) per hexagonal unit cell” rule can be used as a guideline for the synthesis of stable Cu/SAPO-34 catalysts.”.

Some technical issues:
1) The authors should comment on the humidity of “typical ambient lab environment” (Page 4) for sample storage.

We thank the reviewer for the comment. Lab temperature and relative humidity information has been added and highlighted on page 4 of the revised manuscript.

2) Method for the quantification of EPR active Cu species using solution Cu-imide complexes is not described in ref. 19 (Page 6). The authors should check if they cited a wrong reference.

We thank the reviewer for pointing out this mistake. This citation has been removed. In the experimental section, more details regarding EPR measurements have been added (highlighted on pages 20-21 that reads “The isolated Cu(II) contents were quantified using the following method. Typically, spectra acquired at 125 K were first double-integrated to obtain signal areas, which are proportional to EPR active isolated Cu(II) contents. To quantify these, a series of standard solutions with different isolated Cu(II) concentrations were prepared by dissolving $Cu(NO_3)_2·2.5H_2O$ and imidazole (Sigma Aldrich, 99%) in ethylene glycol (Sigma Aldrich, 99.8%). Imidazole was used here to coordinate with Cu(II) ions to prevent formation of EPR silent Cu dimers. The linear calibration curve generated using the integral areas and Cu contents of the standard solutions was then used for quantification of isolated Cu(II) in the catalysts.”

3) Fig. S7/S8: the reviewer suggests to label the different features with dashed lines crossing the peaks for self-explaining of the figures.

We do not quite understand this suggestion. The high-field features are readily distinguished by using different colors to plot different spectra. For the hyperfine region, we already used an insert to magnify that and used dashed lines to show difference between Cu1-F and Cu1-S in Figure S7. If we mark all hyperfine features, this region will become very crowded. For Figure S8, the hyperfine features are already readily distinguished. We don’t think it is necessary to label more.

4) NH_3-TPD/H_2-TPR should be defined upon first appearance in the main article.

The definition of NH_3-TPD/H_2-TPR has been added and highlighted on pages 6, 7 of the revised manuscript.

REVIEWERS' COMMENTS:

Reviewer #1 (Remarks to the Author):

Authors addresses the comments and questions quite well. So further review is not necessary.

Reviewer #3 (Remarks to the Author):

The authors have addressed all the concerns from the reviewers, and the revised manuscript now can be accepted for publication in its current form.
